# An ETHYLENE INSENSITIVE3-LIKE1 Protein Directly Targets the *GEG* Promoter and Mediates Ethylene-Induced Ray Petal Elongation in *Gerbera hybrida*

**DOI:** 10.3389/fpls.2019.01737

**Published:** 2020-01-24

**Authors:** Gan Huang, Meixiang Han, Lin Jian, Yanbo Chen, Shulan Sun, Xiaojing Wang, Yaqin Wang

**Affiliations:** Guangdong Provincial Key Laboratory of Biotechnology for Plant Development School of Life Sciences South China Normal University, Guangzhou, China

**Keywords:** GhEIL1, ethylene, petal elongation, *GEG*, *Gerbera hybrida*

## Abstract

Petal morphogenesis has a profound influence on the quality of ornamental flowers. Most current research on petal development focuses on the early developmental stage, and little is known about the late developmental stage. Previously, it was reported that the *GEG* gene [a gerbera homolog of the gibberellin-stimulated transcript 1 (*GAST1*) from tomato] negatively regulates ray petal growth during the late stage of development by inhibiting longitudinal cell expansion. To explore the molecular mechanisms of the role of *GEG* in petal growth inhibition, an ethylene insensitive 3-like 1 (EIL1) protein was identified from a *Gerbera hybrida* cDNA library by yeast one-hybrid screening. Direct binding between GhEIL1 and the *GEG* promoter was confirmed by electrophoretic mobility shift and dual-luciferase assays. The expression profiles of *GhEIL1* and *GEG* were correlated during petal development, while a transient transformation assay suggested that GhEIL1 regulates *GEG* expression and may be involved in the inhibition of ray petal elongation and cell elongation. To study the effect of ethylene on ray petal growth, a hormone treatment assay was performed in detached ray petals. The results showed that petal elongation is limited and promoted by ACC and 1-MCP, respectively, and the expression of *GhEIL1* and *GEG* is regulated and coordinated during this process. Taken together, our research suggests that GhEIL1 forms part of the ethylene signaling pathway and activates *GEG* to regulate ray petal growth during the late developmental stage in *G. hybrida*.

## Introduction

The arrangement of inflorescences has an important role for plants during the reproductive growth period. The enormous variation in flower shape and structure among species has fascinated scientists for centuries, and it is key to the evolutionary success of angiosperms. The shape and size of flowers also largely affect the ornamental value of plants, especially of cut flowers. Gerbera, of which *Gerbera hybrida* is one of the most important commercially grown varieties, belongs to the large sunflower family (Asteraceae). It has an inflorescence typically consisting of three different types of florets, which from outside to the inside are ray florets, trans florets, and disc florets. This head-like inflorescence structure is very different from that of the classical model plant *Arabidopsis*, and *G. hybrida* is regarded as a new model for floral organ developmental studies, particularly among the Asteraceae (Shepard and Purugganan, 2002; Buzgo et al., 2004; Zhang et al., 2017).

Most recent studies in gerbera focus on flower organ identity. Based on the ABC model of flower development, the B and C orthologs in gerbera control the development of petal, stamen, and carpel, with functions that are largely shared with those in *Arabidopsis* (Yu et al., 1999; Broholm et al., 2010). *GSQUA2*, a putative A function gene, which is involved in floral transition, has also been discovered in *G. hybrida* (Yu et al., 1999; Ruokolainen et al., 2010). The SEP-like genes, *GRCD1* and *GRCD2*, regulate stamen and carpel identities, respectively, and function like E genes in gerbera. Full loss of *GRCD* activities leads to conversion of flower organs into leaves, indicating that *GRCD* genes affect organ identity in a whorl-specific manner (Kotilainen et al., 2000; Uimari et al., 2004; Zhang et al., 2017). After the flower organs are formed, the final size of petals is determined by both cell division and expansion (Mizukami and Fischer, 2000; Szécsi et al., 2006). Some genes influencing floral organ size have been identified in *Arabidopsis*. For example, *ARGOS*, *ANT*, *OSR1*, and *JAGGED* are thought to promote organ growth, as loss of function of these genes results in smaller organs with decreased cell numbers due to the reduced duration of cell proliferation (Mizukami and Fischer, 2000; Hu et al., 2003; Feng et al., 2011). By contrast, *BIG BROTHER* (*BB*), *DA1*, and *DA2* restrict organ size by limiting cell proliferation (Disch et al., 2006; Li et al., 2008; Peng et al., 2013). In addition, *ANGUSTIFOLIA* (*AN*), *ARGOS-LIKE* (*ARL*), and *KUODA1* (*KUA1*) enhance organ growth by promoting cell expansion (Kim et al., 2002; Hu et al., 2006; Liu et al., 2013; Feng et al., 2015). In recent years, several other genes, such as *SPIKE1* (Ren et al., 2016) and *IPGA1* (Yang et al., 2019), have also been implicated in the regulation of petal shape by affecting cell expansion at the late developmental stage. In gerbera, the CYC-like genes play major roles in the regulation of organ growth, both as positive and negative regulators of cell proliferation and/or expansion. For instance, *GhCYC2*, *GhCYC3*, and *GhCYC4* influence the differentiation and growth of ray flowers by affecting cell proliferation (Broholm et al., 2008; Tähtiharju et al., 2011), while *GhCYC5* is probably involved in the expansion of the capitulum by modulating flower initiation (Juntheikki-Palovaara et al., 2014).

Phytohormones are well-known mediators of floral organ growth. In *Arabidopsis*, *BIGPETALp* (*BPEp*) controls petal size by restricting cell expansion; its expression is regulated by jasmonic acid (JA), suggesting that *BPEp* is involved in petal growth and may be mediated by JA (Szécsi et al., 2006; Brioudes et al., 2009; Varaud et al., 2011). Auxin participates in many aspects of floral growth, e.g., ARF8 (auxin response factor 8) negatively regulates petal growth by affecting cell expansion (Aloni et al., 2006; Varaud et al., 2011). Previously, we reported that gibberellin (GA) and abscisic acid (ABA) regulate cell expansion of gerbera petals in an antagonistic manner (Li et al., 2015). GhWIP2, a WIP-type ZFP transcription factor (TF), which is activated by ABA and inhibited by GA, was found to be associated with cell expansion in gerbera (Ren et al., 2018). Another class of plant hormones, brassinosteroids (BRs), promotes petal growth by regulating cell expansion in gerbera. Transcriptome analysis showed that various TFs are activated by brassinolide 0.5 h after treatment, whereas cell wall protein genes are regulated at 10 h (Huang et al., 2017).

Ethylene is reported to be an important regulator of multiple aspects of plant growth and development (Bleecker and Kende, 2000; Zhao and Guo, 2011). For example, the petiole elongates rapidly after ethylene treatment in *Arabidopsis*, due to cell expansion (Bleecker and Kende, 2000). In rose petals, ethylene inhibites cell expansion and water absorption by repressing the expression of *RhPIP2;1* (Ma et al., 2008), while RhNAC100, an ethylene-responsive NAC-domain TF, suppresses petal growth by modulating cell expansion in rose (Pei et al., 2013), indicating that ethylene plays a key role in petal growth. The ethylene signaling pathway begins with ethylene binding to its receptors (ETR1, ERS1, ETR2, EIN4, and ERS2). After binding, the signal is transmited to the downstream component CTR1. The ethylene receptors interact with CTR1 and positively regulate its activity (Kieber et al., 1993; Gao et al., 2003). CTR1 in turn directly or indirectly inhibits EIN2. Subsequently, the EIN2 protein translocates into the nucleus, where it influences the stabilization and activation of the primary TF, EIN3 (Ju and Chang, 2012). Six genes [*AtEIN3* and *AtEIL1–5* (*EIN3-like*)] in the *Arabidopsis* genome belong to the EIN3 family, of which *AtEIL1* is closely related to *AtEIN3* (Guo and Ecker, 2004). *AtEIN3* and *AtEIL1* are the two master TFs that specifically target the promoters of ethylene-response genes and activate or repress their expression, thereby modulating ethylene responses in plants (Boutrot et al., 2010; Zhang et al., 2011; Chang et al., 2013). Overexpression of *AtEIN3* significantly inhibits hypocotyl elongation in the dark (An et al., 2010). The *TEIL* (*Tobacco EIN3-Like*) gene is a tobacco homolog of *AtEIN3*. In *35S*::*TEIL* plants, the pistil length of the flower is longer than wild type (WT), with a slight protrusion of the stigma. In TEIL-suppressed plants, there is a significant protrusion of the stigma and the stamens are shorter than WT, indicating that TEIL is involved in flower organ development in tobacco (Rieu et al., 2003; Hibi et al., 2007; Wawrzynska et al., 2010).

GAST1 was the first GA-stimulated gene identified in plants (Shi et al., 1992). Subsequently, a family of genes encoding proteins containing a GASA (GA-stimulated in *Arabidopsis*) domain was identified in *Arabidopsis* (Herzog et al., 1995). GASA family proteins usually have a conserved C-terminal region of about 60 amino acids that contains 12 cysteine residues in fixed positions (Roxrud et al., 2007). These genes are involved in various developmental processes in plants, such as flowering and stem growth (Ben-Nissan et al., 2004; de la Fuente et al., 2006). *GEG*, a gerbera homolog of the *GAST1* gene, which is induced by GA, suppresses petal growth during the late developmental stage (Kotilainen et al., 1999) and is regulated by upstream TF GhMIF and other putative proteins (Han et al., 2017). However, the molecular mechanism by which *GEG* inhibits petal elongation is still not fully understood. Here, using a yeast one-hybrid (Y1H) screening system, we identified an EIN3-like protein, named GhEIL1, from a *G. hybrida* cDNA library. Using both an electrophoretic mobility shift assay (EMSA) and a dual-luciferase reporter assay, direct binding of GhEIL1 to the EIN3-binding sites in the *GEG* promoter was confirmed. Further studies indicated that GhEIL1 was an EIN3/EIL family protein that might act as a transcriptional regulator to suppress ray petal elongation, probably by modulating *GEG* expression. Moreover, the expression of *GhEIL1* and *GEG* was coordinated during ray petal development and was upregulated by 1-aminocyclopropane-1-carboxylate (ACC) treatment. Taken together, our results indicate that, in response to ethylene, GhEIL1 probably activates *GEG* and participates in the process of ray petal elongation as a negative regulator.

## Materials and Methods

### Plant Materials and Growth Conditions

A cultivar of *G. hybrida* named ‘Linglong’ was used in this study. Individual shoots were grown in multiplication medium to obtain bud clusters and were then kept in a tissue-culture room at 24 ± 2°C. After rooting, the plants were transferred to a greenhouse grown at a temperature of 24 ± 2°C and relative humidity of 65–80%. The flower developmental stages were based on Meng and Wang (2004). Inflorescences at stage 3 were used for transient transformation and hormone treatments.

*Arabidopsis thaliana* (Columbia ecotype) seeds were sown on plates with MS medium. After germination, the plates were transferred to a tissue-culture room under long-day conditions (16-h light/8-h dark). One week later, the seedlings were transferred to a phytotron under long-day conditions at 24 ± 2°C.

### Cloning and Sequence Analysis of *GhEIL1*

A full-length *GhEIL1* cDNA was amplified from a gerbera cDNA library by 5′-RACE and reverse transcription PCR (RT-PCR) as previously described (Liu et al., 2015). Alignment of the deduced amino acid sequences with EIN3 homologues in different species was performed using ClustalX 1.83 and DNAMAN 7.0 (default values were used), and phylogenetic analysis was performed using ClustalX 1.83 and MEGA 6.0. The phylogenetic trees were computed using the neighbor-joining algorithm with 10,000 bootstrap replicates. The primers used in this study are listed in **Table S1**.

### Subcellular Localization

Protoplasts were isolated from gerbera leaves as described previously (Ren et al., 2018). YFP-GhEIL1 [GhEIL1 fused to yellow fluorescence protein (YFP) driven by the *CaMV35S* promoter] and YFP empty vector (YFP driven by the *CaMV35S* promoter) were transfected into protoplasts. The nuclear localization marker NLS-mCherry (a construct with the nuclear localization signal fused to the mCherry protein) (Li et al., 2019) was co-transfected into gerbera protoplasts to label the nucleus. Fluorescence analysis was performed using a laser confocal microscope (LSM710, Carl Zeiss, Germany).

### Dual-Luciferase Reporter Assay

To study whether GhEIL1 binds to the *GEG* promoter, reporter vector pGREEN0800-LUC [which contains the renilla luciferase (REN) reporter gene driven by the *CaMV35S* promoter and the firefly luciferase (LUC) reporter gene with multiple cloning sites (MCS) upstream for insertion of different candidate promoter fragments as needed] and effector vector pBluescript [which contains a mirabilis mosaic virus (*MMV*) promoter and the *rbcS* terminator] were used. The experiment was performed as follows.

First, recombinant reporter plasmids were constructed. Based on the position and number of EIN3-binding sites (EBs) in the *GEG* promoter, the promoter was divided into various fragments named *proGEG260*, *proGEG402*, *proGEG468*, and *proGEG975* (shown in **Figure 2D**). Three putative EBs (TACAT) were found in the −580 to −260-bp region of the promoter (Zhong et al., 2009), and therefore this region was cloned and named *proGEG320* (−580 to −261). Mutated versions of *proGEG320* (shown in **Figures 2C**, **E**) were also used in this experiment. These fragments were inserted into the MCS of pGREEN0800-LUC using various restriction enzymes to generate the reporter vectors. Second, full-length *GhEIL1* was inserted into the MCS of the pBS vector to generate the effector plasmid. Third, pBS-GhEIL1 was co-transformed with the various reporter plasmids into gerbera protoplasts. To characterize ethylene-responsive activity, the protoplasts were treated with 10 µM ACC after transformation. The empty pBS vector (EV) was co-transformed with different reporters as no-interaction controls. Finally, following the above transformations, protoplast samples were incubated at 24–26°C overnight, then harvested and analyzed using the dual-luciferase reporter assay system with a VeritasTM Microplate Luminometer (Promega, Madison, WI, USA) (Yoo et al., 2007; Ren et al., 2018).

To analyze GhEIL1 transcriptional activity, full-length *GhEIL1* was inserted into the modified pBS vector [which contains the *MMV* promoter, five copies of the GAL4 DNA binding domain (GALBD), and the *rbcS* terminator] to act as effector. The reporter vector contains a minimal *CaMV35S* promoter with five tandem copies of the GAL4 response element (GALRE) upstream, a firefly LUC gene, and the *rbcS* terminator (Pattanaik et al., 2006). The renilla luciferase (REN) gene, regulated by the *CaMV35S* promoter and the *rbcS* terminator, was used as internal control. Schematic representations of these constructs are shown in **Figure 3C**. All three types of plasmid (effector, reporter, and internal control vector) were co-transformed into gerbera protoplasts. The protoplast incubation conditions and determination of fluorescence activity were as described above. Three biological replicates were used for each experiment.

### Yeast One-Hybrid Screen

To investigate the binding between GhEIL1 and the *GEG* promoter, *proGEG320* and *proGEG260* were inserted into the pAbAi vector as bait sequences, while full-length *GhEIL1* cDNA was inserted into the pGADT7 vector to provide the prey protein. Y1H assays were performed according to the user manual (Matchmaker Gold Yeast One-Hybrid Library Screening System, cat. no. 630491; Clontech, United States). Plates were incubated for 3 days at 30°C, after which yeast growth was assessed.

### Yeast Two-Hybrid System

Full-length *GhEIL1* cDNA was subcloned into the pGBKT7 vector to generate the BD-GhEIL1 construct. The BD-GhEIL1 vector and empty pGADT7 vector were co-transformed into the AH109 yeast strain (Gietz and Schiestl, 2007), which contains four reporter genes (*HIS3*, *ADE2*, *MEL1*, and *lacZ*), to test the transcriptional activation ability of GhEIL1. Transformation was performed according to the manufacturer’s instructions (Matchmaker® Gold Yeast Two-Hybrid System, cat. no. 630489; Clontech, United States). Synthetic dropout medium (SD/-Leu-Trp-His) was used to select the positive clone. X-α-gal (5-bromo-4-chloro-3-indolyl-d-galactopyranoside; Clontech) is a substrate of α-galactosidase (encoded by the *MEL1* gene). When X-α-gal is hydrolyzed by α-galactosidase, a blue product is formed. X-α-gal was added to synthetic dropout medium for high-stringency screening.

### Electrophoretic Mobility Shift Assay

The EMSA was performed as described (Han et al., 2017). Briefly, a recombinant pET28a-SUMO-GhEIL1 plasmid was transformed into *Escherichia coli* BL21 cells. After incubation at 22°C overnight, cells were harvested and lysed, then the recombinant protein was purified on Ni-NTA columns (Qiagen, Germany). The *proGEG320* probe and the complementary probe were labelled with biotin. About 1 mg purified recombinant protein and 50 nM biotin-labeled probe were used for each sample. The experiment was performed using a lightshift chemiluminescent EMSA kit (Thermo Scientific, United States).

### Transient Transformation of Ray Petals

Ray petal transformation experiments in this study consisted of transient overexpression and virus-induced gene silencing (VIGS). Full-length *GhEIL1* cDNA was subcloned into the pCanG vector under the control of the *CaMV35S* promoter and a nopaline synthase (*nos*) terminator (Yin et al., 2009; Ren et al., 2018). A gene-specific fragment of *GhEIL1* (386 bp in length) was also used to construct the vector pTRV2-GhEIL1. Then, pTRV1, pTRV2, pTRV2-GhEIL1, and pCanG-GhEIL1 were separately transformed into *Agrobacterium tumefaciens* strain GV3101. Next, 5 ml Luria–Bertani (LB) medium supplemented with 50 mg ml^−1^ kanamycin and 100 mg ml^−1^ rifampicin was inoculated with each *A. tumefaciens* strain and shaken at 220 rpm and 28°C overnight. The cultures were then each inoculated into 50 ml LB medium supplemented with 20 µM acetosyringone (AS) and 10 mM 2-(N-morpholino) ethanesulfonic acid (MES) and shaken at 28°C overnight. Bacterial cultures were harvested and resuspended in infiltration buffer (200 µM AS, 10 mM MES, and 10 mM MgCl_2_, pH 5.6) to a final absorbance (OD_600_) of 1.2. *A. tumefaciens* cultures carrying pTRV2-GhEIL1 and pTRV1 were mixed at a ratio of 1:1 (*v*/*v*), while pTRV2 and pTRV1 cultures were mixed at the same ratio as a negative control. The mixtures of pTRV2-GhEIL1/pTRV1, pTRV2/pTRV1, and pCanG-GhEIL1 (resuspended in infiltration buffer as above at OD_600_ = 1.2) were stored in the dark for 4–6 h at room temperature.

For the transient transformation assay, detached ray petals (~2.0-cm lengths) were used. Fresh inflorescences at the same stage were picked in the greenhouse, and the ray petals were detached and cleaned with sterile distilled water (ddH_2_O). The cleaned petals were then submerged in different buffers as mentioned above and exposed to a vacuum of −0.09 MPa for 5 min. The infiltrated petals were washed several times with ddH_2_O and placed in a sterile plastic Petri dish with filter papers soaked in ddH_2_O. After incubation at 4°C for 3 days, the petals were transferred to a growth chamber at 24–26°C for 8 days (Pei et al., 2013; Han et al., 2017). At least 20 well-developed inflorescences were used for each treatment, and at least three biological replicates were used for each experiment.

### Genetic Transformation of *Arabidopsis*

To obtain transgenic lines, genetic transformation of *Arabidopsis* was performed following the *A. tumefaciens-*mediated floral dipping transformation method described previously (Su et al., 2016).

### Hormone Treatment of Ray Petals

Detached petals from inflorescences were used for hormone treatments as described previously (Huang et al., 2017). Ray petals were detached from the inflorescences at stage 3, then cleaned and wiped gently as described above. One hundred micromolar ACC (a precursor that can be rapidly converted to ethylene by plants) or 50 µM aminoethoxyvinyl glycine (AVG, a competitive inhibitor of ACS enzyme that suppresses endogenous ACC biosynthesis) was used for treatment. 1-Methylcyclopropene (1-MCP) binds ethylene receptors irreversibly and inhibits ethylene action. The 1-MCP treatment method used is similar to that described by Hussain et al. (2019). 1-MCP was obtained in granules (Shanghai Yuanye Biological Technology Co. Ltd.) and released by dissolving in water; it was maintained at 4°C until needed. At temperatures above 6.8°C, the 1-MCP gas was released from the solution. The final concentration of 1-MCP used in this study was 100 mg/m^3^. For hormone treatment, the cleaned petals were placed in square sterile plastic Petri dishes (10 × 10 × 1.5 cm^3^) on two layers of Whatman filter paper soaked in the various diluted solutions mentioned above. Then the Petri dishes were covered and sealed with parafilm to maintain humidity. Subsequently, the plates were transferred to a growth chamber at 24–26°C for 6 days. Petals treated with ddH_2_O were used as control. At least 20 well-grown inflorescences were used for each treatment, and at least three biological replicates were used for each experiment.

### Measurement of Petal and Cell Length

To measure petal length, a total of 60 petals for each treatment were collected. Images of petals were captured using an Epson-G850A scanner (Epson, China) and their lengths were measured using ImageJ software (http://rsb.info.nih.gov/ij/; NIH, MD, USA).

To measure cell length, a block of petal tissue of about 1 mm^2^ from the basal region of a ray petal was dissected and stained by immersion in 0.1 mg/ml propidium iodide for 30 min, then rinsed and tiled on a glass slide. Images of adaxial epidermal cells were captured using a confocal scanning microscope (Carl-Zeiss, Germany). The morphological characteristics of adaxial epidermal cells and abaxial epidermal cells in gerbera petals are shown in **Figure S3**. Abaxial epidermal cells are much longer and thinner, and the cell contour is more irregular, compared with the adaxial epidermal cells. There are greater differences in length between different abaxial epidermal cells, and stomata on the abaxial surface can affect the observations. Therefore, we usually chose adaxial epidermal cells for cell length observation and measurement. More than 100 cells were randomly selected from each treatment and were measured using ImageJ. All data were analyzed as described previously (Li et al., 2015). At least three biological replicates were used for each observation and measurement.

### Measurement of Ethylene Content

To measure ethylene production by petals in the presence of ACC, 1 g fresh petal sample (at stage 3, which was used for hormone treatments) was submerged in 1 mM ACC in a 10-ml gas chromatography vial. Petals in ddH_2_O and ACC solution without petals were used as negative controls. The vials were incubated at 30°C for 1 h, then ethylene content was measured using an Agilent Technologies 7890A gas chromatography system with a capillary column (Agilent Technologies, USA).

### Quantitative Real-Time PCR

Total RNA was extracted according to the user manual (MiniBEST Universal RNA Extraction Kit, cat. no. 9767; TaKaRa, Japan). For quantitative real-time PCR (qRT-PCR), the method was described previously (Su et al., 2016). Gene expression levels were normalized to that of the *GhACTIN* (AJ763915) gene, as previously described (Kuang et al., 2013). Each qRT-PCR experiment was repeated at least three times.

## Results

### Cloning and Analysis of GhEIL1 in *G. hybrida*

**Figure 1:**
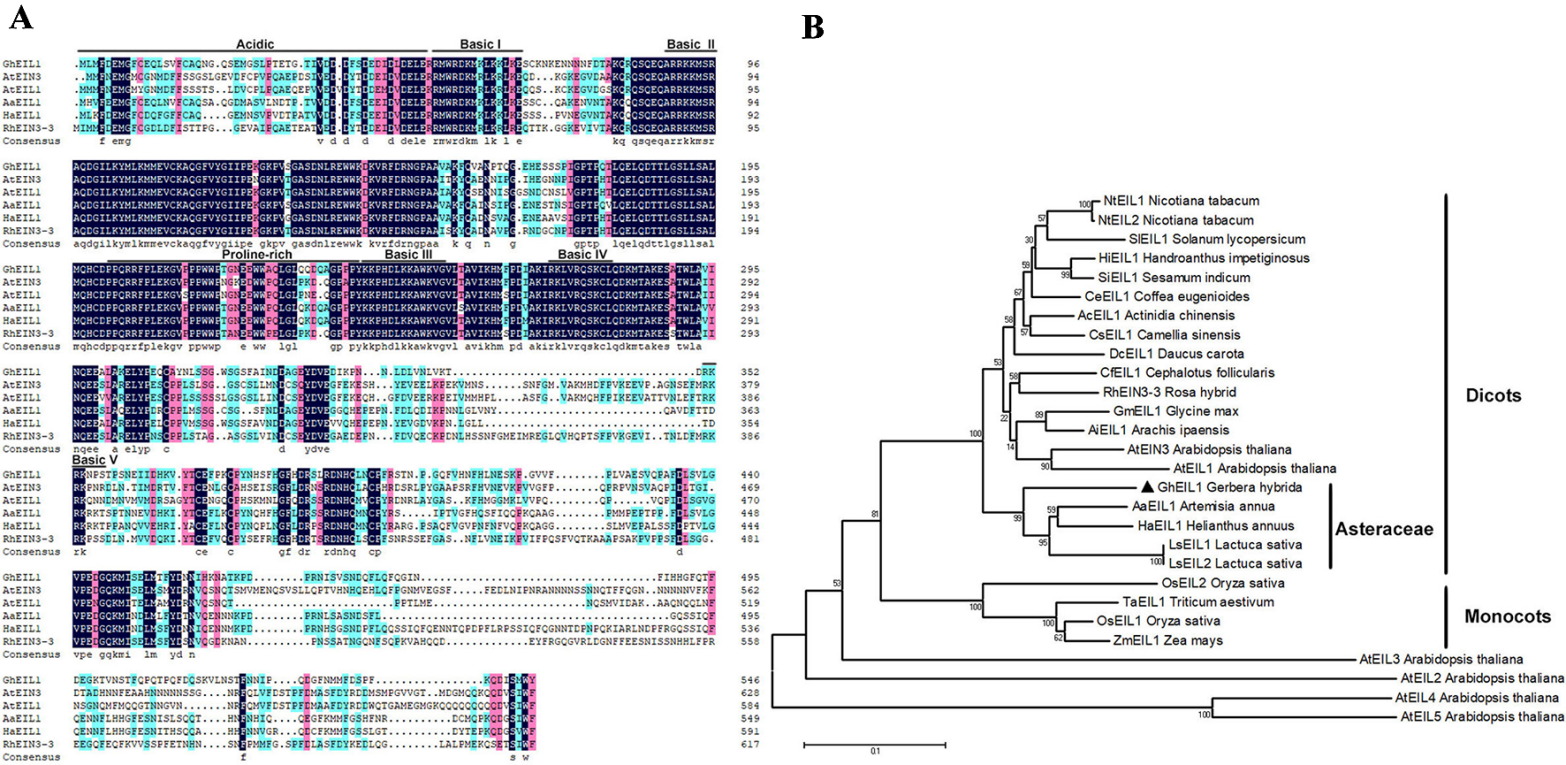
Analysis of EIL1 protein sequences. **(A)** Amino acid sequence alignment of the EIL1 proteins of various plant species. Acidic regions, proline-rich regions, and basic domains are represented by *black lines above the sequences*. **(B)** Phylogeny of the EIN3-like family genes in different species. The phylogenetic tree was constructed with MEGA 5.1 using the neighbor-joining method. The bootstrap values shown indicate the robustness of each branch. The *scale bar* represents 0.1 substitutions per site. AaEIL1 (PWA97162.1) *Artemisia annua*; LsEIL1 (XP_023744606.1) *Lactuca sativa*; HaEIL1 (XP_022006414.1) *Helianthus annuus*; LsEIL2 (PLY65573.1) *Lactuca sativa*; AcEIL1 (PSS14565.1) *Actinidia chinensis*; DcEIL1 (XP_017252734.1) *Daucus carota*; CsEIL1 (XP_028062292.1) *Camellia sinensis*; HiEIL1 (PIN12929.1) *Handroanthus impetiginosus*; NtEIL1 (NP_001312793.1) *Nicotiana tabacum*; SiEIL1 (XP_011080514.1) *Sesamum indicum*; NtEIL2 (NP_001312850.1) *Nicotiana tabacum*; CeEIL1 (XP_027163785.1) *Coffea eugenioides*; CfEIL1 (GAV86009.1) *Cephalotus follicularis*; GmEIL1 (XP_003555660.1) *Glycine max*; AiEIL1 (XP_016166039.1) *Arachis ipaensis*; RhEIN3-3 (AGK07288.1) *Rosa hybrid*; SlEIL1 (NP_001234541.1) *Solanum lycopersicum*; OsEIL1 (XP_015629857.1) *Oryza sativa*; OsEIL2 (XP_015646574.1) *Oryza sativa*; TaEIL1 (AMW92184.1) *Triticum aestivum*; ZmEIL1 (NP_001152035.2) *Zea mays*; AtEIN3 (AT3G20770.1) *Arabidopsis thaliana*; AtEIL1(AT2G27050) *Arabidopsis thaliana;* AtEIL2 (AT5G21120) *Arabidopsis thaliana*; AtEIL3 (AT1G73730) *Arabidopsis thaliana*; AtEIL4 (AT5G10120) *Arabidopsis thaliana*; AtEIL5 (AT5G65100) *Arabidopsis thaliana*.

*GEG* (AJ005206) suppresses ray petal growth at the late stage of *G. hybrida* development (Kotilainen et al., 1999). In our previous study, we cloned the *GEG* promoter and identified a mini zinc-finger protein (GhMIF) that can bind to the *GEG* promoter directly (Han et al., 2017). In this paper, we initially identified a sequence encoding an ethylene insensitive 3-like protein (GhEIL1) using a Y1H screen. A full-length GhEIL1 cDNA was cloned by reverse transcription PCR (RT-PCR) and 5′-RACE, and contained a 1,641-bp open reading frame that encoded a protein of 547 amino acids; its nucleotide sequence was deposited in GenBank (MF370883). To investigate the biological role of GhEIL1, we identified and aligned the protein sequences of EIN3/EIL family members from various species. GhEIL1 shared 53% identity with AtEIN3 and AtEIL1 from *Arabidopsis*. As shown in **Figure 1A**, the amino-terminal half of GhEIL1 is more conserved than the carboxy-terminal half. The sequences of basic domains and BD I–IV show significant similarity, while the acidic, proline-rich regions and BD V are highly divergent compared with those of other species (**Figure 1A**). Such acidic and proline-rich regions are widely regarded as transcriptional activation domains (Mermod et al., 1989; Mitchell and Tjian, 1989; Chao et al., 1997). Subsequent phylogenetic tree analysis revealed the evolutionary relationship between GhEIL1 and other EIN3-like proteins. As shown in **Figure 1B**, GhEIL1 clustered together with EIN3-like proteins from other Asteraceae family species like *Artemisia annua*, *Lactuca sativa*, and *Helianthus annuus*. The Asteraceae group also includes examples from other dicotyledonous species, for example, AtEIN3 and AtEIL1 from *Arabidopsis*. Intriguingly, other *Arabidopsis* EIL proteins are highly divergent, as previously reported (Feng et al., 2017). This analysis suggests that GhEIL1 is a homolog of AtEIN3 and AtEIL1, similarly to EIN3-like proteins from other Asteraceae family species, and thus may have a similar function.

### Direct Binding of GhEIL1 to EIN3-Binding Sites in the GEG Promoter

**Figure 2:**
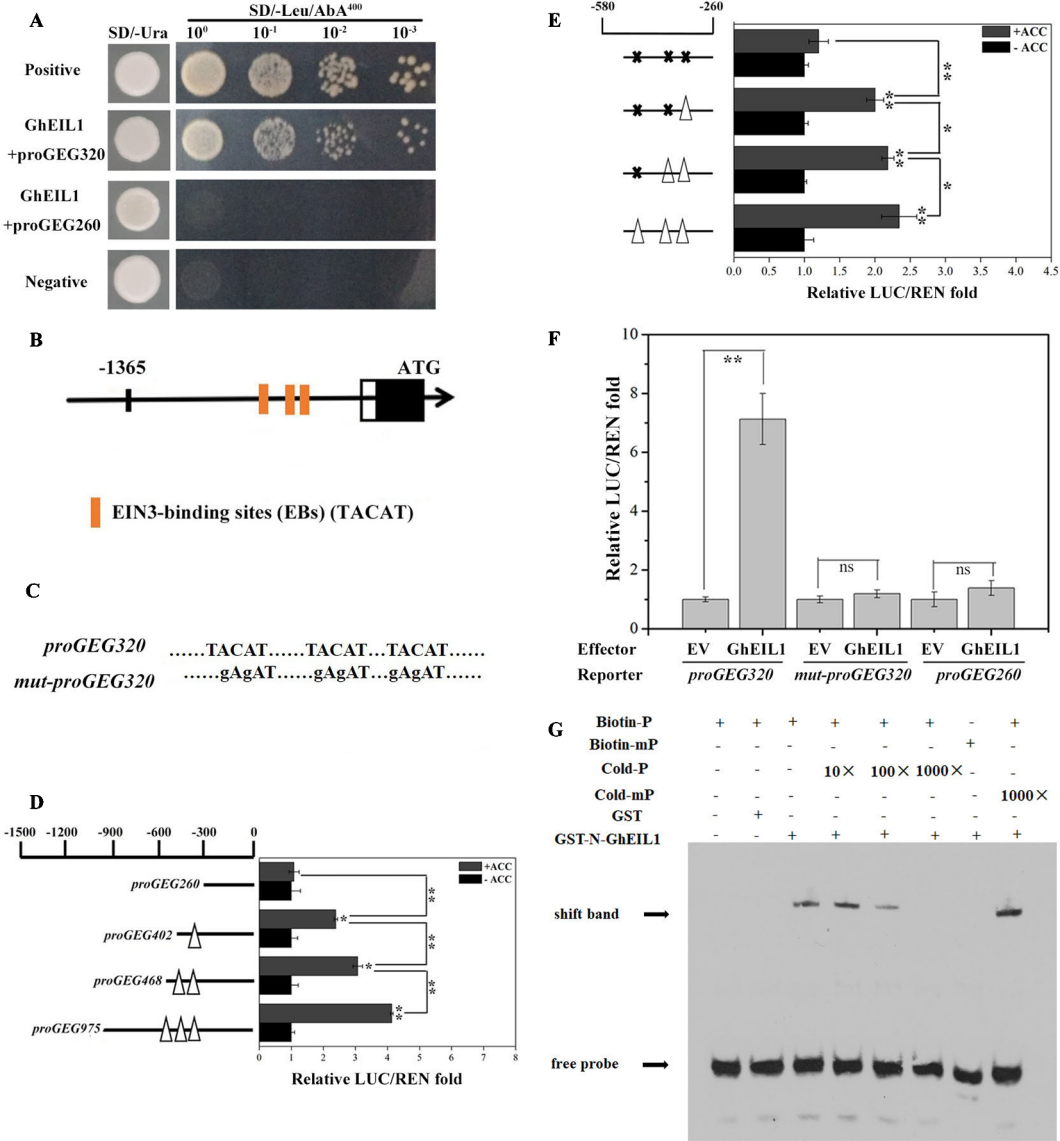
Activity analysis of EIN3-binding sites in the *GEG* promoter and the direct binding of GhEIL1 to the *GEG* promoter. **(A)** Yeast one-hybrid analysis showing the interaction between GhEIL1 and the *GEG* promoter. pGADT7-Rec-p53/p53-AbAi and pGADT7/proGEG320 were used as positive and negative controls, respectively. GhEIL1/proGEG260 was used as the non-specific binding control. 10^0^, 10^−1^, 10^−2^, and 10^−3^ represent the fold dilution of bacteria. **(B)** Three distinctive EIN3-binding sites were predicted in the promoter region of the *GEG*. **(C)** Schematic representation of a version of the promoter fragment in which the EIN3-binding site (EB) elements were mutated. **(D)** Dual-luciferase assay indicating the responses of four truncated *GEG* promoter fragments containing different EBs to 1-aminocyclopropane-1-carboxylate (ACC) treatment *in vivo*. **(E)** Dual-luciferase assay indicating the responses of site-directed mutated versions of the *GEG* promoter to ACC treatment *in vivo*. *Empty triangles* and *solid crosses* represent EBs before and after mutation, respectively. **(F)** Dual-luciferase assay indicating the interaction between GhEIL1 and the *GEG* promoter *in vivo*. pBS-GhEIL1 (full-length *GhEIL1* cDNA was fused to the pBS vector in the MCS, driven by a *MMV* promoter) was used as an effector, and *proGEG320* was inserted in pGREEN0800-LUC as a reporter. The reporter vectors containing *proGEG260* and mut-*proGEG320* were co-transformed with pBS-GhEIL1 as non-specific binding controls. The empty pBS vector (EV) was co-transformed with these reporters as no-interaction controls. The GhEIL1/*proGEG260* and GhEIL1/mut-*proGEG320* constructs exhibited a LUC/REN ratio similar to that of their respective controls (EV/*proGEG260* and EV/mut-*proGEG320*). The LUC/REN of the control was set to 1.0. Values are the means ± SD from three biological replicates. **(G)** EMSA analysis showing binding of GhEIL1 to the *GEG* promoter. The *black arrow* indicates the binding of GhEIL1 and the biotin-labeled GEG probe. The + and − signs represent the presence and absence of corresponding components, respectively. Significant differences were determined using ANOVA and Tukey’s HSD: *p < 0.05, **p < 0.01.

We identified GhEIL1 from the gerbera cDNA library using a bait construct containing *proGEG320* in a Y1H assay. The interaction site between GhEIL1 and *GEG* was confirmed because yeast transformed with *GhEIL1* and *proGEG320* grew in the SD selection medium (lacking Leu and containing 400 ng/ml AbA), while yeast transformed with *GhEIL1* and *proGEG260* did not (**Figure 2A**). These results are consistent with specific binding of GhEIL1 to the *GEG* promoter.

Three putative EBs (TACAT) were found in the −580 to −260-bp region of the promoter (Zhong et al., 2009) (**Figure 2B**). To investigate whether these EBs are involved in the response to ethylene, we carried out a dual-luciferase reporter assay using *GEG* promoter truncations with different numbers of EBs. As shown in **Figure 2D**, in the presence of ACC, the LUC/REN ratio reduced from 4.03 to 1.08 as promoter length and number of EBs decreased. The LUC/REN ratio was similar to the control (i.e., in the absence of ACC) when all EBs were deleted. We then performed this experiment using versions of the promoter in which the putative EB elements were progressively mutated. Although mutation of each of the two most distal EBs produced only a small decrease in LUC/REN ratio, the largest decrease was observed when all three elements were mutated; in the latter case, there was no significant difference in LUC/REN ratio whether ACC was present or not (**Figures 2C**, **E**). These results suggest that the EBs in the promoter are important for the *GEG* response to ethylene treatment.

Three versions of the reporter construct, containing either *proGEG320*, *mut-proGEG320* (**Figure 2C**), or *proGEG260* (the *GEG* promoter region from −260 to 0 bp, which lacks EB elements), were co-introduced into gerbera protoplasts with either an effector construct containing GhEIL1 or EV. Co-transformation of *proGEG320* and GhEIL1 resulted in an extremely high LUC/REN ratio compared with the EV control, while the LUC/REN ratios for the *mut-proGEG320* and *proGEG260* reporter constructs were similar to controls (**Figure 2F**). These results indicate that GhEIL1 can specifically bind to the EB region of the *GEG* promoter *in vivo* and can activate the expression of a downstream gene.

To confirm direct binding between GhEIL1 and the *GEG* promoter, EMSA analysis was performed. Biotin-labeled probes were designed according to the core *GEG* promoter region *proGEG320*, while a probe corresponding to the same region, but without biotin, was used as a competitor. The results showed that GhEIL1 was able to bind to the biotin-labeled *proGEG320* probe, but did not bind when the EBs were mutated. Furthermore, this binding was gradually attenuated by increasing the concentration of unlabeled probe, but was not outcompeted by the mutated probe (**Figure 2G**). This suggests that GhEIL1 binds directly to the *GEG* promoter by specific interaction with its EB elements.

### GhEIL1 is a Transcription Activator and Coordinates With *GEG* During Petal Growth

**Figure 3:**
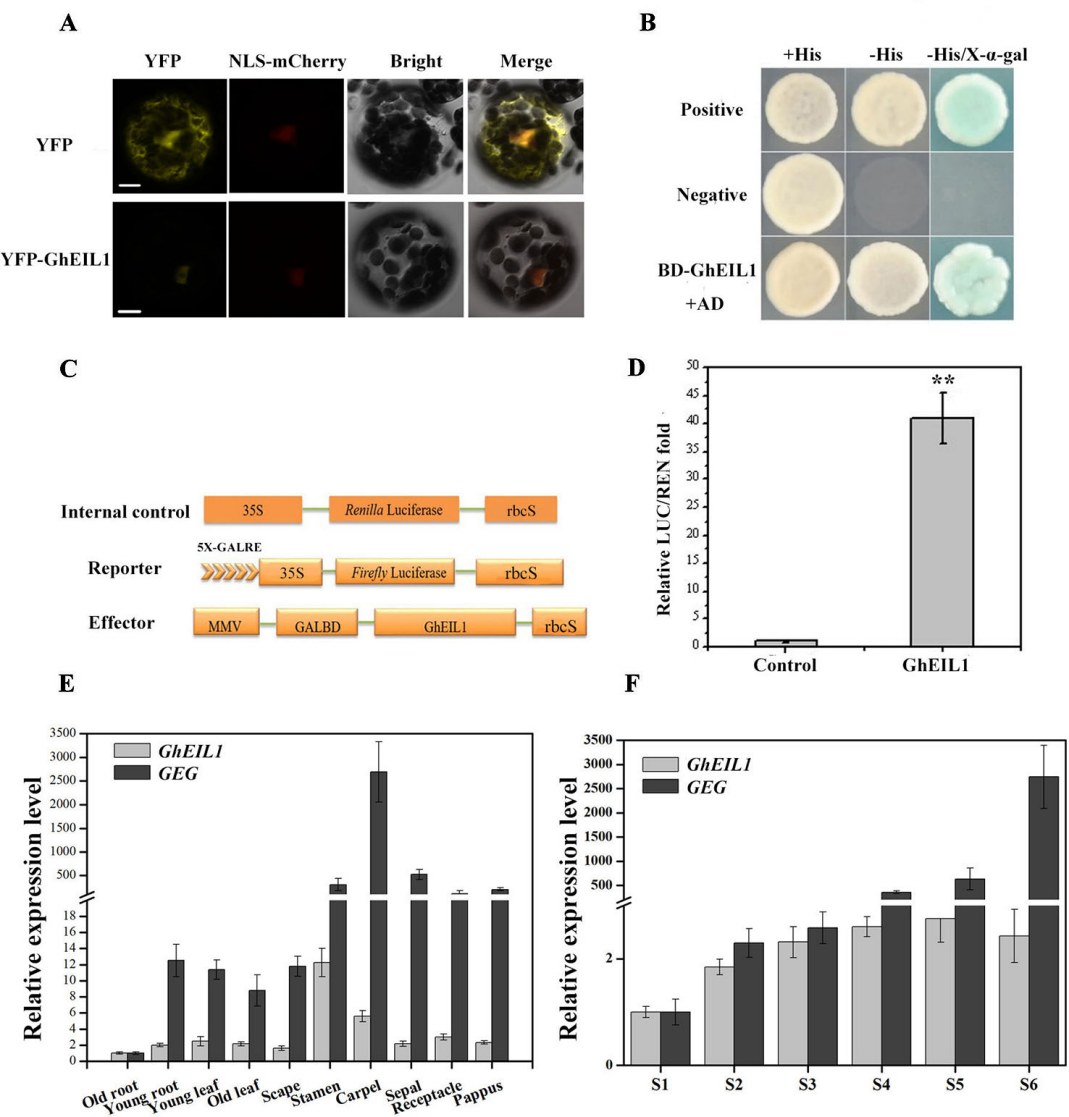
Subcellular localization and transcription activity of the GhEIL1 protein and the expression pattern of *GhEIL1* and *GEG*. **(A)** Subcellular localization of the GhEIL1 protein in gerbera protoplasts. YFP protein driven by the 35S promoter was transformed as a control. NLS-mCherry was co-transformed as nuclear marker. *Bars*, 10 µm. **(B)** Analysis of the transcriptional activation activity of GhEIL1 using yeast one-hybrid assay. *AD*, pGADT7 with activation domain; *BD*, pGBKT7 with binding domain; positive control, pGBKT7-p53 transformed with pGADT7-SV40 large T antigen; negative control, empty pGBKT7 vector transformed with pGADT7 vector. **(C)** Schematic representation of the constructs used in the dual-luciferase assay. **(D)** Relative transcriptional activity of GhEIL1 using dual-luciferase assay. The experimental group (GhEIL1) was performed by co-transforming the vectors shown in **(C)**; the modified empty pBS vector was co-transformed with reporter and internal control constructs as the control. **(E)** The relative expression level of *GEG* and *GhEIL1* during petal developmental phases (S1–S6, “S” represents “stage”) and in different tissues and floral organs of the ray petals **(F)**. Values are the means ± SD from three biological replicates. ** indicates a significant difference at *p* < 0.01.

We determined the subcellular localization of the GhEIL1 protein by expressing a YFP-GhEIL1 fusion protein in gerbera mesophyll protoplasts. A NLS-mCherry construct was co-transformed into protoplasts as a nuclear marker. As shown in **Figure 3A**, the YFP-GhEIL1 fusion protein was found only in the nucleus, while YFP protein alone was detected in both cytoplasm and nucleus.

To investigate the effect of GhEIL1 on transcription of a target gene, the yeast two-hybrid (Y2H) system and a dual-luciferase assay were used. For the Y2H experiment, BD-GhEIL1 and pGADT7 (AD) constructs were co-transformed into yeast strain AH109. As shown in **Figure 3B**, the resultant yeast cells were able to grow on synthetic dropout medium (SD/-Leu-Trp-His), and the medium became blue when X-α-gal was added. These results indicate that GhEIL1 might be a transcriptional activator. For the dual-luciferase reporter assay, *GhEIL1* was inserted into the modified pBS vector as an effector, while the reporter construct contained a firefly LUC gene driven by a minimal *CaMV35S* promoter with five tandem copies of GALRE positioned upstream; *Renilla* luciferase (REN) driven by the *CaMV35S* promoter was used as internal control. A schematic representation of the constructs is shown in **Figure 3C**. The empty pBS vector with reporter and internal control (without the effector construct) were co-transformed as a negative control. The REN/LUC ratio of the experimental group was significantly higher than the control (**Figure 3D**). Taken together, these results indicate that GhEIL1 acts as a transcriptional activator of *GEG*.

We next investigated the expression of *GhEIL1* and *GEG* in different organs and tissues in gerbera and found that both genes were expressed in all organs, but with a relatively high level of expression in the floral organs and the highest expression level in stamen and carpel (**Figure 3E**). We also analyzed the expression of *GhEIL1* during the various growth stages of ray petals and observed that *GhEIL1* expression levels gradually increased from stage 1 to stage 5, and then decreased slightly in stage 6, while *GEG* expression increased continuously from stage 1 to stage 6 (**Figure 3F**). The similar expression profiles of *GhEIL1* and *GEG* suggest that their expression might be coordinated during petal growth.

### GhEIL1 Inhibits Ray Petal Elongation by Regulating *GEG* Expression

**Figure 4:**
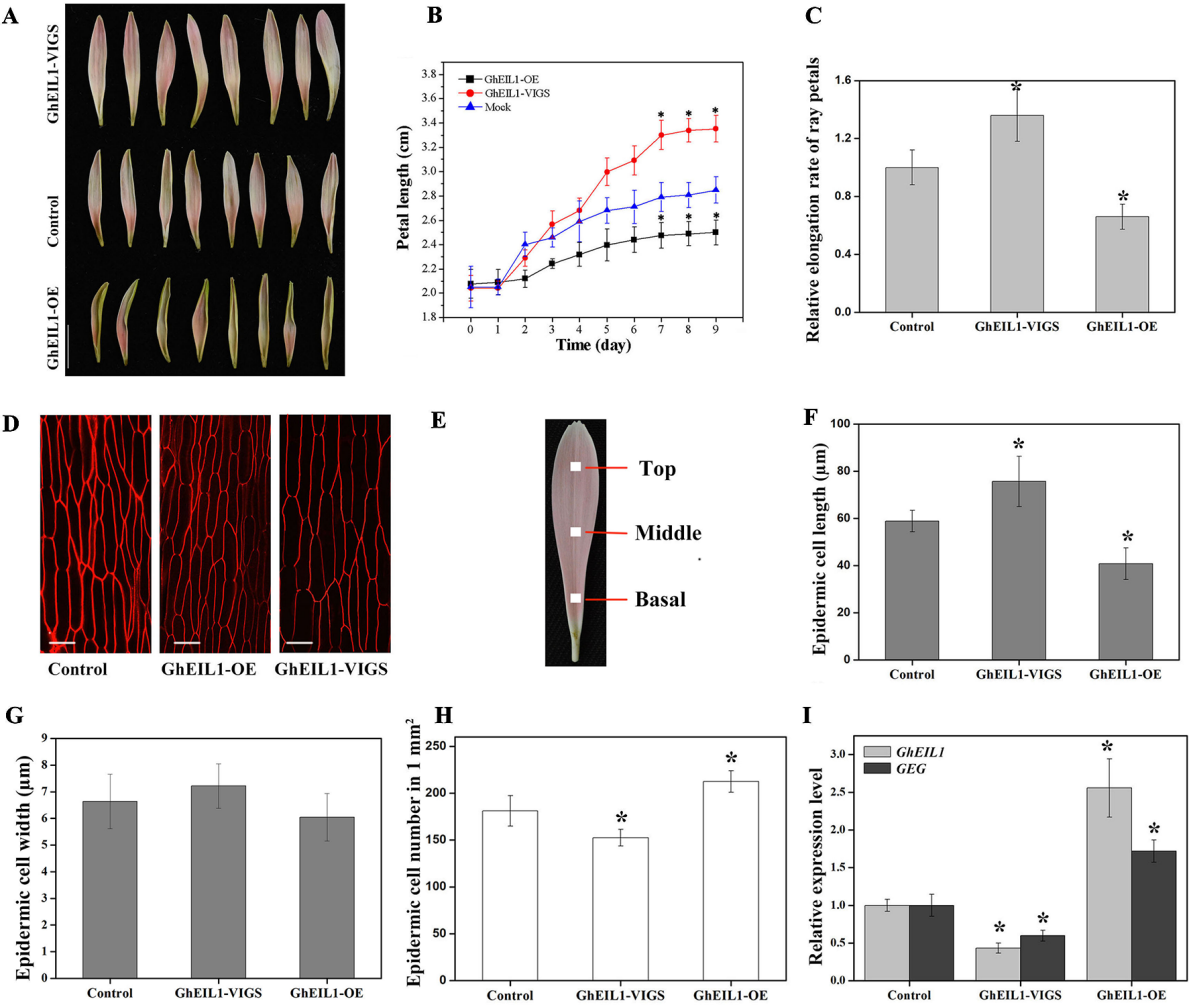
Petals with transiently overexpressed *GhEIL1* or after virus-induced gene silencing (VIGS) in *G. hybrida*. **(A)** Phenotypes of GhEIL1-VIGS, GhEIL1-OE, and control petals. **(B)** Time-course dynamics of petal length in control, GhEIL1-VIGS, and GhEIL1-OE petals after transformation (*n* = 30). **(C)** Relative elongation rate of ray petals. **(D)** Morphological characterization of adaxial epidermal cells in the basal region of control, GhEIL1-OE, and GhEIL1-VIGS petals. **(E)** Blocks (1 mm^2^) at the center of the basal, middle, and top regions of ray petals were sampled for morphological characterization of petal cells. Cell length **(F)**, cell width **(G)**, and cell number **(H)** of control, GhEIL1-VIGS, and GhEIL1-OE petals in the basal region. **(I)** Expression level of *GhEIL1* and *GEG* in control, GhEIL1-VIGS, and GhEIL1-OE petals. Experimental petals were collected after 9 days cultivation. *Scale bars* represent 1 cm **(A)** or 20 µm **(D)**. All values indicate means ± SD from at least three biological replicates. Significant differences were determined using ANOVA and Tukey’s HSD: *p < 0.05.

To study the function of *GhEIL1* during petal growth, transient transformation assays were performed in detached gerbera petals. VIGS was carried out to suppress *GhEIL1* expression using the natural defense mechanisms of plants. A transient overexpression assay was performed by vacuum infiltration of the *A. tumefaciens* strain carrying GhEIL1 under the control of the *CaMV35S* promoter. After transformation with either GhEIL1-overexpressing (GhEIL1-OE) or GhEIL1-silencing (GhEIL1-VIGS) constructs, the petals were kept at 4°C for 3 days, and then transferred to a tissue-culture room at room temperature for 9 days. After that, the *GhEIL1* and *GEG* expression levels and petal lengths were measured. We found that petal elongation was substantially promoted in GhEIL1-VIGS petals and inhibited in GhEIL1-OE petals (**Figure 4A**). Thus, the average petal length was 3.4 ± 0.3 cm in GhEIL1-VIGS samples and 2.5 ± 0.2 cm in GhEIL1-OE samples compared with 2.9 ± 0.2 cm in the control. The relative elongation rate of petals was 1.36 in silenced samples and 0.66 in overexpressing samples compared with controls (**Figures 4B, C**).

Final petal size is determined by both cell division and cell elongation. To test which of these predominates, the number, length, and width of the cells in ray petals were measured. Each petal was divided into three parts for these observations (**Figure 4E**). In our previous study, it was found that the basal region of ray petals was the main zone of elongation (Li et al., 2015), so we mainly focused on this area. As shown in **Figures 4D**, **F**, the average length of the epidermal cells was 75.7 ± 10.7 µm in GhEIL1-VIGS petals and 40.8 ± 6.7 µm in GhEIL1-OE petals compared with 58.9 ± 4.5 µm in the controls. The cell width slightly decreased in GhEIL1-OE petals (6.0 ± 60.9 µm) and slightly increased in GhEIL1-VIGS petals (7.1 ± 0.8 µm) compared with the control (6.6 ± 1.0 µm), but the differences were not statistically significant (**Figure 4G**). The average cell number in a 1-mm^2^ area of petal was 181.3 ± 16.2 in controls, 152.5 ± 8.8 in silenced petals, and 212.5 ± 11.6 in overexpressing petals (**Figure 4H**). Together, these data suggest that *GhEIL1* inhibits petal elongation at least partly by a role in repression of cell elongation in gerbera ray petals. In addition, the cells in the middle and top regions of ray petals were also measured. As shown in **Figure S2A**, the cell length showed a similar trend to that of the basal region, but the difference was not significant.

To determine the expression levels of *GhEIL1* and *GEG* in the transiently transformed petals, qRT-PCR was used. We found that the *GhEIL1* expression level was significantly increased (∼2.50-fold) in the overexpressed samples and decreased (∼0.47-fold) in the silenced samples compared with the control (**Figure 4I**). In addition, *GEG* expression was significantly increased (∼1.76-fold) in GhEIL1-OE petals, but decreased (∼0.56-fold) in GhEIL1-VIGS petals (**Figure 4I**). These results suggest that GhEIL1 inhibits petal growth by regulating the expression of *GEG*.

### GhEIL1 Involvement in Ethylene-Inhibited Petal Elongation is Partly Due to its Effect on *GEG* Expression

**Figure 5:**
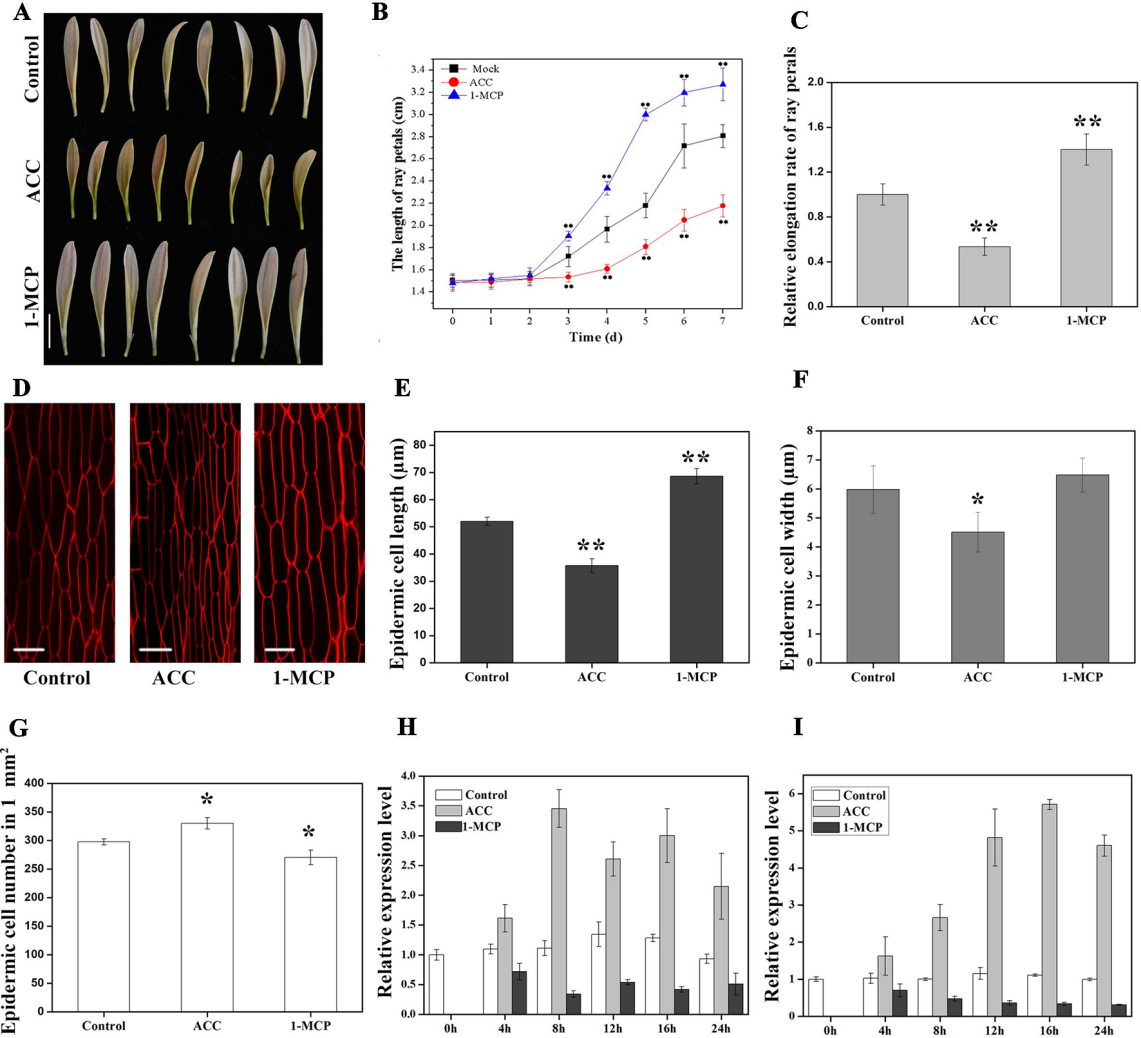
Effects of 1-aminocyclopropane-1-carboxylate (ACC) and 1-methylcyclopropene (1-MCP) on petal growth in *G. hybrida*. **(A)** Phenotypes of petals treated with deionized water (control), ACC, and 1-MCP for 7 days. **(B)** Time-course dynamics of petal length under control conditions or after treatment with ACC or 1-MCP. A total of 30 petals for each treatment were cultured for 7 days. **(C)** Relative elongation rate of ray petals after 7-day treatments. **(D)** Characterization of adaxial epidermal cells in the basal region after control, ACC, and 1-MCP treatments. Cell length **(E)**, cell width **(F)**, and cell number **(G)** of petals after control, ACC, and 1-MCP treatments in the basal region (*n* > 100). Expression level of *GhEIL1* **(H)** and *GEG* **(I)** in ray petals after control, ACC, and 1-MCP treatments at different time points (0, 4, 8, 12, 16, and 24 h). Three biological replicates were performed for each measurement. *Scale bar* represents 1 cm **(A)** or 20 µm **(D)**. All values indicate means ± SD. Significant differences were evaluated by ANOVA and Tukey’s HSD test: *p < 0.05, **p < 0.01.

Ethylene is a key regulator in plant growth and development. In order to examine the role of ethylene in petal growth, hormone treatment experiments were carried out using ray petals at stage 3. We found that ACC could be converted to ethylene in gerbera petals at stage 3 (**Figure S4**), and therefore ACC was used in the following experiment. After 7 days of ACC treatment, petal length was significantly suppressed compared with controls, with average lengths of 2.18 ± 0.14 cm and 2.81 ± 0.22 cm, respectively; in contrast, average petal length was 3.27 ± 0.28 cm in 1-MCP-treated samples, i.e., these petals were significantly longer than controls (**Figures 5A**, **B**). The relative elongation rate of petals following ACC treatment was 0.54 and, following 1-MCP treatment, 1.40, compared with controls (**Figure 5C**). To investigate the effect of reducing ethylene levels, we treated gerbera petals with AVG, a competitive inhibitor of the enzyme, ACS. As shown in **Figure S5**, the change in petal length was similar between AVG and 1-MCP treatments, although the trend was weaker with AVG. Nevertheless, these results indicate that ethylene inhibits petal growth in gerbera.

We next examined cell size in the basal region of the petal. The average length of epidermal cells was 68.6 ± 2.8 µm in 1-MCP-treated petals and 35.7 ± 2.6 µm in ACC-treated petals compared with 52.0 ± 1.5 µm in the control (**Figures 5D**, **E**). Cell elongation rates were 36% in the presence of ACC, 57% following 1-MCP treatment, and 45% in the control. Cell width decreased in ACC-treated petals (4.5 ± 0.7 µm) compared with controls (6.0 ± 0.8 µm), while there was no significant difference in cell width in 1-MCP-treated petals (6.4 ± 0.6 µm) (**Figure 5F**). However, the average cell number in a 1-mm^2^ area of petal showed the opposite trend to the cell length (**Figures 5E**, **G**). Cells in the middle and top regions were also measured after treatments. As shown in **Figure S2B**, the change in cell length seemed similar to that of the basal region, albeit without showing a significant difference. Together, these results indicate that ethylene suppresses ray petal growth at least in part by reducing cell elongation.

EIN3 is known as the primary TF in ethylene signaling (Zhao and Guo, 2011). In order to examine whether GhEIL1 is involved in ethylene signaling, the *GhEIL1* gene was ectopically overexpressed in *Arabidopsis*. Three independent lines (OE2, OE4, and OE9) with a highly expressed *GhEIL1* gene were chosen for further study. As shown in **Figure S1**, in the absence of ACC, the transgenic plants behaved like WT. However, in the presence of ACC, the growth of transgenic seedlings was significantly suppressed compared with WT, indicating that *GhEIL1* may affect plant growth *via* the ethylene signaling pathway.

To test whether GhEIL1 is involved in the process by which ethylene influences petal growth, we determined the expression levels of *GhEIL1* and *GEG* in gerbera petals following treatment with ACC or 1-MCP. The results showed that the expression of *GhEIL1* and *GEG* was upregulated after ACC treatment and downregulated in the presence of 1-MCP over a 24-h period (**Figures 5H**, **I**). Thus, *GhEIL1* and *GEG* respond to ethylene in a similar manner, implicating GhEIL1 in the process by which ethylene inhibits petal growth and suggesting that this is, at least in part, due to its effect on *GEG* expression.

## Discussion

### GhEIL1 Inhibits Ray Petal Elongation by Directly Targeting the *GEG* Promoter and Regulating *GEG* Expression in *G. hybrida*

Ethylene plays important roles in cell expansion during plant growth. EIN3 and EIL1 are two major TFs in the ethylene signaling pathway. Overexpressing *EIL1* in the *ein3 ebf1 ebf2* triple mutant results in flowers with stunted petals and protruding gynoecia, indicating that *EIL1* is important for flower growth in *Arabidopsis* (An et al., 2010). In this study, a homolog of *EIN3*, *GhEIL1*, was identified in gerbera. GhEIL1 shares 53% identity with AtEIN3 and AtEIL1. GhEIL1 also contains conserved domains found in EIN3/EIL family proteins (**Figure 1**). These findings suggest that GhEIL1 has a similar function in the ethylene signaling pathway in *G. hybrida* to that in other plants. Subcellular localization experiments showed that GhEIL1 is only found in the nucleus (**Figure 3A**), which is consistent with GhEIL1 having transcriptional activation activity, as demonstrated in yeast and gerbera protoplasts (**Figures 3B**, **D**). It thus seems likely that GhEIL1 works as a transcription activator in *G. hybrida*, similarly to AtEIN3 in *Arabidopsis* (Chao et al., 1997; Solano et al., 1998).

*GhEIL1* was identified from a gerbera cDNA library using a bait construct containing the *GEG* promoter. Using Y1H, dual-luciferase reporter assay, and EMSA, we confirmed that GhEIL1 acts as an upstream regulator of *GEG* by directly binding to the *GEG* promoter at EIN3-binding sites (**Figures 2A**, **F**, **G**). Further study showed that GhEIL1 was highly expressed in the flower organs and was upregulated during ray petal elongation from stage 1 to stage 6 (**Figures 3E**, **F**), indicating that *GhEIL1* is involved in petal growth as well as petal elongation. A transient transformation assay was used to investigate the effect of *GhEIL1* on petal growth and showed that the elongation rate increased by 66% in GhEIL1-VIGS petals and decreased by 50% in GhEIL1-OE petals (**Figures 4A**, **B**); thus, GhEIL1 is a negative regulator of petal elongation in gerbera. Furthermore, *GEG* was significantly upregulated in petals that overexpressed *GhEIL1* and was downregulated in those with silenced *GhEIL1*. These data suggest that GhEIL1 inhibits ray petal elongation at least partly by regulating *GEG* expression (**Figure 2**).

Petal development after floral primordia formation and differentiation depends on cell division and cell expansion (Alvarez-Buylla et al., 2010; Krizek and Anderson, 2013). It has been reported that petal size in gerbera is mainly determined by cell expansion at stage 3 (Meng and Wang, 2004; Laitinen et al., 2007; Zhang et al., 2012). Our study showed that the cell length in the basal region of ray petals is clearly longer in GhEIL1-VIGS petals compared with controls, suggesting that *GhEIL1* inhibits ray petal elongation at least partly by affecting cell elongation, in agreement with previous studies (Li et al., 2015; Huang et al., 2017).

*GEG*, the first GAST1-like gene found in gerbera, plays a role in the regulation of cell shape during corolla and carpel development and is mainly expressed in the later developmental stages of petal growth (Kotilainen et al., 1999). Another GAST1-like gene, *PRGL* (proline-rich and GASA-like), is highly expressed in the early stages of petal development and may promote petal growth (Peng et al., 2008; Peng et al., 2010). These reports indicate that GASA family genes play important roles in petal development in gerbera, but the mechanisms of regulation are not well characterized. In our previous study, we found that a TF GhMIF acts as a transcriptional activator by directly binding to the *GEG* promoter (Han et al., 2017). The current work shows that GhEIL1 also acts as a TF and activates *GEG* by directly binding to the *GEG* promoter at the EIN3-binding sites (**Figures 2A**, **F**, **G**). A number of other proteins, like GhBZR1 (MF370884) and GhMBF1 (MF370886), were also identified previously using a Y1H screen (Han et al., 2017), suggesting that *GEG* is regulated by multiple upstream TFs in *G. hybrida*.

### Petal Growth is Regulated by a Complex Network of Plant Hormone Pathways

Petal growth is a complex process controlled by gene regulatory networks that modulate cell proliferation and cell expansion, and thereby determine the final size and shape of petals. Plant hormones play important roles in petal growth. In our previous study in gerbera, we found GA and ABA to have opposite effects on petal size determination (Li et al., 2015). A WIP-type zinc finger protein, named GhWIP2, was found to be involved in this process. Overexpression of *GhWIP2* in gerbera results in major developmental defects, including dwarfism, short petals, short scapes, and short petioles (Ren et al., 2018). BRs are also known to promote petal growth by lengthening cells in gerbera, and their effect is greater than that of GA (Huang et al., 2017).

In rose, ethylene accelerates the flower-opening process by suppressing the expansion of petal cells, thereby further suppressing petal expansion (Ma et al., 2008). In addition to its effect on petal cells, ethylene clearly suppresses petal growth in rose, while the ethylene synthesis inhibitor 1-MCP increases petal length and cell size. In this study, petal length and cell elongation were reduced by ACC treatment, while the opposite situation pertained after 1-MCP treatment (**Figure 5**). These findings strongly suggest that reduced cell expansion may contribute to the inhibition of petal expansion by ethylene in gerbera, in accordance with observations in rose.

RhNAC100, a homolog of AtNAC2 in rose, was found to function as a negative regulator of cell expansion in rose petal (Pei et al., 2013). In gerbera, GhEIL1 inhibits ray petal elongation by regulating *GEG* expression, as discussed above. We also found that ethylene increases the expression of *GhEIL1* and *GEG* (**Figure 5**), suggesting that *GhEIL1* and *GEG* are regulated directly by ethylene. Additionally, *GhEIL1*-overexpressing petals exhibit similar morphological and anatomical phenotypes to petals treated with ethylene, while *GhEIL1*-silenced petals showed the opposite phenotypes (**Figure 4**), further supporting the notion that ethylene inhibits petal expansion in gerbera by activating *GhEIL1* expression. The combined results suggest that GhEIL1 can respond to ethylene and is involved in ethylene-regulated petal expansion.

Recent research has provided some insights into the crosstalk between the hormones that regulate petal growth. For example, *BPEp*, whose expression is regulated by JA, controls petal size by restricting cell expansion, indicating that *BPEp* may be involved in JA-mediated petal growth (Szécsi et al., 2006; Varaud et al., 2011). BPEp also interacts with ARF8, which is a protein in the auxin signaling pathway that inhibits petal expansion. In a *bpe arf8* double mutant, petal size is significantly increased compared with WT, as well as with the *bpe-1* and *arf8-3* single mutants. Together, the above data suggest that crosstalk between auxin and JA occurs during the regulation of petal growth in *Arabidopsis* (Szécsi et al., 2006; Varaud et al., 2011). *RhEIN3-3*, an EIN3-like gene in rose, directly targets the promoter of *RhGAI1* (an ethylene-responsive DELLA gene). In addition, *RhGAI1* is involved in cell expansion by regulating the expression of several cell expansion-related genes, suggesting the occurrence of crosstalk between ethylene and GA during rose petal growth (Luo et al., 2013). *GEG* inhibits petal elongation in gerbera and responds to GA treatment (Kotilainen et al., 1999). We found *GEG* to be upregulated by ethylene treatment and targeted by GhEIL1, which indicates that there is also crosstalk between ethylene and GA during the regulation of petal growth in gerbera.

In summary, our research suggests that petal growth is a complex process probably regulated by a series of different hormones, like ethylene, GA, BR, and ABA, and involves crosstalk between them. Various plant hormones control genes, such as *GhWIP2* and *GhMIF* in the GA pathway, and *GhEIL1* in the ethylene pathway, to influence petal growth in gerbera. This research lays the foundations for further studies into the mechanism of action and interaction of the various hormones involved in controlling petal growth in gerbera.

## Data Availability Statement

The gene sequences used in this study can be found in NCBI (https://www.ncbi.nlm.nih.gov/): GEG (AJ005206), GhEIL1(MF370883). The datasets generated for this study are available on request to the corresponding author.

## Author Contributions

GH and YW carried out the experiments and drafted the manuscript. GH, MH, XW, and LJ analyzed the data. XW and YC participated in figure preparation. SS and XW participated in experimental design and manuscript. YW conceived the study, participated in its design and manuscript revision. All authors read and approved the final manuscript.

## Funding

This work was supported by National Key R&D Program of China (2018YFD1000404), National Natural Science Foundation of China (31672188, 31700282, 31601784, 31572161), Science and Technology Plan Project of Guangdong Province (2017A030312004, 2016A020208013, 2015B020231009).

## Conflict of Interest

The authors declare that the research was conducted in the absence of any commercial or financial relationships that could be construed as a potential conflict of interest.

## References

[B1] Aloni R. Aloni E. Langhans M. Ullrich C. (2006). Role of cytokinin and auxin in shaping root architecture: regulating vascular differentiation, lateral root initiation, root apical dominance and root gravitropism. Ann. Bot. 97, 883–893. 10.1093/aob/mcl027 16473866 PMC2803412

[B2] Alvarez-Buylla E. R. Benítez M. Corvera-Poiré A. Cador Á. C. de Folter S. de Buen A. G. (2010). Flower development. Arabidopsis Book 8, e0127. 10.1199/tab.0127 22303253 PMC3244948

[B3] An F. Zhao Q. Ji Y. Li W. Jiang Z. Yu X. (2010). Ethylene-induced stabilization of ETHYLENE INSENSITIVE3 and EIN3-LIKE1 is mediated by proteasomal degradation of EIN3 binding F-Box 1 and 2 that requires EIN2 in Arabidopsis. Plant Cell 22, 2384–2401. 10.1105/tpc.110.076588 20647342 PMC2929093

[B4] Ben-Nissan G. Lee J. Y. Borohov A. Weiss D. (2004). GIP, a Petunia hybrida GA-induced cysteine-rich protein: a possible role in shoot elongation and transition to flowering. Plant J. 37, 229–238. 10.1046/j.1365-313x.2003.01950.x 14690507

[B5] Bleecker A. B. Kende H. (2000). Ethylene: a gaseous signal molecule in plants. Annu. Rev. Cell Dev. Biol. 16, 1–18. 10.1146/annurev.cellbio.16.1.1 11031228

[B6] Boutrot F. Segonzac C. Chang K. N. Qiao H. Ecker J. R. Zipfel C. (2010). Direct transcriptional control of the *Arabidopsis* immune receptor FLS2 by the ethylene-dependent transcription factors EIN3 and EIL1. Proc. Natl. Acad. Sci. U.S.A. 107, 14502–14507. 10.1073/pnas.1003347107 20663954 PMC2922558

[B7] Brioudes F. Joly C. Szécsi J. Varaud E. Leroux J. Bellvert F. (2009). Jasmonate controls late development stages of petal growth in *Arabidopsis thaliana*. Plant J. 60, 1070–1080. 10.1111/j.1365-313x.2009.04023.x 19765234

[B9] Broholm S. K. Tähtiharju S. Laitinen R. A. E. Albert V. A. Teeri T. H. Elomaa P. (2008). A TCP domain transcription factor controls flower type specification along the radial axis of the Gerbera (Asteraceae) inflorescence. Proc. Natl. Acad. Sci. U.S.A. 105, 9117–9122. 10.1073/pnas.0801359105 18574149 PMC2449374

[B8] Broholm S. K. Pollanen E. Ruokolainen S. Tahtiharju S. Kotilainen M. Albert V. A. (2010). Functional characterization of B class MADS-box transcription factors in Gerbera hybrida. J. Exp. Bot. 61, 75–85. 10.1093/jxb/erp279 19767305 PMC2791112

[B10] Buzgo M. Soltis. P. S. Soltis D. E. (2004). Floral developmental morphology of Amborella trichopoda (Amborellaceae). Int. J. Plant Sci. 165, 925–947. 10.1086/424024

[B11] Chang K. N. Zhong S. Weirauch M. T. Hon G. Pelizzola M. Li H. (2013). Temporal transcriptional response to ethylene gas drives growth hormone cross-regulation in Arabidopsis. Elife 2, e00675. 10.7554/elife.00675 23795294 PMC3679525

[B12] Chao Q. Rothenberg M. Solano R. Roman G. Terzaghi W. Ecker J. R. (1997). Activation of the ethylene gas response pathway in *Arabidopsis* by the nuclear protein ETHYLENE-INSENSITIVE3 and related proteins. Cell 89, 1133. 10.1016/s0092-8674(00)80300-1 9215635

[B13] de la Fuente J. I. Amaya I. Castillejo C. Sánchez-Sevilla J. F. Quesada M. A. Botella M. A. (2006). The strawberry gene FaGAST affects plant growth through inhibition of cell elongation. J. Exp. Bot. 57, 2401–2411. 10.1093/jxb/erj213 16804055

[B14] Disch S. Anastasiou E. Sharma V. K. Laux T. Fletcher J. C. Lenhard M. (2006). The E3 ubiquitin ligase BIG BROTHER controls arabidopsis organ size in a dosage-dependent manner. Curr. Biol. 16, 272–279. 10.1016/j.cub.2005.12.026 16461280

[B15] Feng G. Liu G. Xiao J. (2015). The arabidopsis EIN2 restricts organ growth by retarding cell expansion. Plant Signaling Behav. 10, e1017169. 10.1080/15592324.2015.1017169 PMC4622927 26039475

[B71] Feng G. Qin Z. Yan J. Zhang X. Hu Y. (2011). Arabidopsis ORGAN SIZE RELATED1 regulates organ growth and final organ size in orchestration with ARGOS and ARL. New Phytol. 191, 635–646. 10.1111/j.1469-8137.2011.03710.x 21457262

[B16] Feng Y. Xu P. Li B. Li P. Wen X. An F. (2017). Ethylene promotes root hair growth through coordinated EIN3/EIL1 and RHD6/RSL1 activity in *Arabidopsis*. Proc. Natl. Acad. Sci. 114, 13834–13839. 10.1073/pnas.1711723115 29233944 PMC5748182

[B73] Gao Z. Chen Y. F. Randlett M. D. Zhao X. C. Findell J. L. Kieber J. J. (2003). Localization of the Raf-like kinase CTR1 to the endoplasmic reticulum of Arabidopsis through participation in ethylene receptor signaling complexes. J. Biol. Chem. 278, 34725–34732. 10.1074/jbc.M305548200 12821658

[B17] Gietz R. D. Schiestl R. H. (2007). High-efficiency yeast transformation using the LiAc/SS carrier DNA/PEG method. Nat. Protoc. 2, 31–34. 10.1038/nprot.2007.13. 17401334

[B18] Guo H. Ecker J. R. (2004). The ethylene signaling pathway: new insights. Curr. Opin. Plant Biol. 7, 40–49. 10.1016/j.pbi.2003.11.011 14732440

[B19] Han M. Xuefeng J. Wei Y. Lingjie K. Gan H. Yujin T. (2017). A mini zinc-finger protein (MIF) from Gerbera hybrida activates the GASA protein family gene, GEG, to inhibit ray petal elongation. Front. Plant Sci. 8, 1649. 10.3389/fpls.2017.01649 29018462 PMC5615213

[B20] Herzog M. Dorne A. M. Grellet F. (1995). GASA, a gibberellin-regulated gene family from Arabidopsis thaliana related to the tomato GAST1 gene. Plant Mol. Biol. 27, 743–752. 10.1007/bf00020227 7727751

[B21] Hibi T. Kosugi S. Iwai T. Kawata M. Seo S. Mitsuhara I. (2007). Involvement of EIN3 homologues in basic PR gene expression and flower development in tobacco plants. J. Exp. Bot. 58, 3671–3678. 10.1093/jxb/erm216 17965144

[B22] Hu Y. Poh H. M. Chua N. H. (2006). The *Arabidopsis* ARGOS-LIKE gene regulates cell expansion during organ growth. Plant J. 47, 1–9. 10.3410/f.1033198.375173 16824178

[B72] Hu Y. Xie Q. Chua N. H. (2003). The Arabidopsis auxin-inducible gene ARGOS controls lateral organ size. Plant Cell. 15, 1951–1961. 10.1105/tpc.013557 12953103 PMC181323

[B23] Huang G. Han M. Yao W. Wang Y. (2017). Transcriptome analysis reveals the regulation of brassinosteroids on petal growth in Gerbera hybrida. PeerJ. 5, e3382. 10.7717/peerj.3382 28584713 PMC5455292

[B24] Hussain S. Bai Z. Huang J. Cao X. Zhu L. Zhu C. (2019). 1-Methylcyclopropene modulates physiological, biochemical, and antioxidant responses of rice to different salt stress levels. Front. Plant Sci. 10, 124. 10.3389/fpls.2019.00124 30846992 PMC6393328

[B25] Ju C. Chang C. (2012). Advances in ethylene signaling: protein complexes at the endoplasmic reticulum (ER) membrane. Aob Plants. 2012, pls031. 10.1093/aobpla/pls031 23119138 PMC3485614

[B26] Juntheikki-Palovaara I. Tähtiharju S. Lan T. Broholm S. K. Rijpkema A. S. Ruonala R. (2014). Functional diversification of duplicated CYC2 clade genes in regulation of inflorescence development in G erbera hybrida (Asteraceae). Plant J. 79, 783–796. 10.1111/tpj.12583 24923429

[B74] Kieber J. J. Rothenberg M. Roman G. Feldman K. A. Ecker J. R. (1993). CTR1, a negative regulator of the ethylene response pathway in Arabidopsis, encodes a member of the raf family of protein kinases. Cell 72, 427–441. 10.1016/0092-8674(93)90119-b 8431946

[B27] Kim G. T. Shoda K. Tsuge T. Cho K. H. Uchimiya H. Yokoyama R. (2002). The ANGUSTIFOLIA gene of *Arabidopsis*, a plant CtBP gene, regulates leaf-cell expansion, the arrangement of cortical microtubules in leaf cells and expression of a gene involved in cell-wall formation. EMBO J. 21, 1267–1279. 10.1093/emboj/21.6.1267 11889033 PMC125914

[B29] Kotilainen M. Helariutta Y. Mehto M. Pöllänen E. Albert V. A. Elomaa P. (1999). GEG participates in the regulation of cell and organ shape during corolla and carpel development in Gerbera hybrida. Plant Cell. 11, 1093–1104. 10.2307/3870801 10368180 PMC144246

[B28] Kotilainen M. Elomaa P. Uimari A. Albert V. A. Yu D. Teeri T. H. (2000). GRCD1, an AGL2-Like MADS box gene, participates in the C function during stamen development in Gerbera hybrida. Plant Cell. 12, 1893–1902. 10.1105/tpc.12.10.1893 11041884 PMC149127

[B30] Krizek B. A. Anderson J. T. (2013). Control of flower size. J. Exp. Bot. 64, 1427–1437. 10.1093/jxb/ert025 23404902

[B31] Kuang Q. Li L. Peng J. Sun S. Wang X. (2013). Transcriptome analysis of Gerbera hybrida ray florets: putative genes associated with gibberellin metabolism and signal transduction. PloS One 8, e57715. 10.1371/journal.pone.0057715 23472101 PMC3589416

[B32] Laitinen R. A. Pöllänen E. Teeri T. H. Elomaa P. Kotilainen M. (2007). Transcriptional analysis of petal organogenesis in Gerbera hybrida. Planta. 226, 347–360. 10.1007/s00425-007-0486-2 17334783

[B35] Li Y. Zheng L. Corke F. Smith C. Bevan M. W. (2008). Control of final seed and organ size by the DA1 gene family in *Arabidopsis thaliana*. Genes Dev. 22, 1331–1336. 10.3410/f.1109351.565383 18483219 PMC2377187

[B34] Li L. Zhang W. Zhang L. Li N. Peng J. Wang Y. (2015). Transcriptomic insights into antagonistic effects of gibberellin and abscisic acid on petal growth in Gerbera hybrida. Front. Plant Sci. 6, 168. 10.3389/fpls.2015.00168 25852718 PMC4362084

[B33] Li H. Li Y. Zhao Q. Li T. Wei J. Li B. (2019). The plant ESCRT component FREE1 shuttles to the nucleus to attenuate abscisic acid signalling. Nat. Plants. 5, 512. 10.1038/s41477-019-0400-5 30962512

[B36] Liu D. Liu X. Meng Y. Sun C. Tang H. Jiang Y. (2013). An organ-specific role for ethylene in rose petal expansion during dehydration and rehydration. J. Exp. Bot. 64, 2333. 10.1093/jxb/ert092 23599274 PMC3654423

[B37] Liu F. Wang X. Su M. Yu M. Zhang S. Lai J. (2015). Functional characterization of DnSIZ1, a SIZ/PIAS-type SUMO E3 ligase from Dendrobium. BMC Plant Biol. 15, 225. 10.1186/s12870-015-0613-3 26376625 PMC4574183

[B38] Luo J. Ma N. Pei H. Chen J. Li J. Gao J. (2013). A DELLA gene, RhGAI1, is a direct target of EIN3 and mediates ethylene-regulated rose petal cell expansion via repressing the expression of RhCesA2. J. Exp. Bot. 64, 5075–5084. 10.1093/jxb/ert296 24014864 PMC3830487

[B39] Ma N. Xue J. Li Y. Liu X. Dai F. Jia W. (2008). Rh-PIP2;1, a rose aquaporin gene, is involved in Ethylene-regulated petal expansion. Plant Physiol. 148, 894–907. 10.1104/pp.108.120154 18715962 PMC2556823

[B40] Meng X. Wang X. (2004). Regulation of Fower development and anthocyanin accumulation in Gerbera hybrida. J. Hortic. Sci. Biotech. 79, 131–137. 10.1111/j.1399-3054.2008.01071.x

[B41] Mermod N. O’Neill E. A. Kelly T. J. Tjian R. (1989). The proline-rich transcriptional activator of CTF/NF-I is distinct from the replication and DNA binding domain. Cell. 58, 741–753. 10.1016/0092-8674(89)90108-6 2504497

[B42] Mitchell P. J. Tjian R. (1989). Transcriptional regulation in mammalian cells by sequence-specific DNA binding proteins. Science 245, 371–378. 10.1126/science.2667136 2667136

[B43] Mizukami Y. Fischer R. L. (2000). Plant organ size control: AINTEGUMENTA regulates growth and cell numbers during organogenesis. Proc. Natl. Acad. Sci. U.S.A. 97, 942–947. 10.1016/s1369-5266(00)80047-3 10639184 PMC15435

[B44] Pattanaik S. Xie C. H. Kong Q. Shen K. A. Yuan L. (2006). Directed evolution of plant basic helix–loop–helix transcription factors for the improvement of transactivational properties. Biochim. Biophys. Acta (BBA)-Gene Struct Expression. 1759, 308–318. 10.1016/j.bbaexp.2006.04.009 16837081

[B45] Pei H. Ma N. Tian J. Luo J. Chen J. Li J. (2013). An NAC transcription factor controls ethylene-regulated cell expansion in flower petals. Plant Physiol. 163, 775–791. 10.1104/pp.113.223388 23933991 PMC3793057

[B46] Peng J. Lai L. Wang X. (2008). PRGL: a cell wall proline-rich protein containning GASA domain in Gerbera hybrida. Sci. Chin Ser. C: Life Sci. 51, 520–525. 10.1007/s11427-008-0067-z 18488172

[B47] Peng J. Lai L. Wang X. (2010). Temporal and spatial expression analysis of PRGL in Gerbera hybrida. Mol. Biol. Rep. 37, 3311–3317. 10.1007/s11033-009-9917-4 19885740

[B48] Peng Y. Ma W. Chen L. Yang L. Li S. Zhao H. (2013). Control of root meristem size by DA1-RELATED PROTEIN2 in *Arabidopsis*. Plant Physiol. 161, 1542–1556. 10.1104/pp.112.210237 23296689 PMC3585615

[B50] Ren H. Dang X. Yang Y. Huang D. Liu M. Gao X. (2016). SPIKE1 activates ROP GTPase to modulate petal growth and shape. Plant Physiol. 172, 358–371. 10.1104/pp.16.00788 27440754 PMC5074625

[B49] Ren G. Li L. Huang Y. Wang Y. Zhang W. Zheng R. (2018). Gh WIP 2, a WIP zinc finger protein, suppresses cell expansion in Gerbera hybrida by mediating crosstalk between gibberellin, abscisic acid, and auxin. New Phytol. 219, 728–742. 10.1111/nph.15175 29681133

[B51] Rieu I. Mariani C. Weterings K. (2003). Expression analysis of five tobacco EIN3 family members in relation to tissue-specific ethylene responses. J. Exp. Bot. 54, 2239–2244. 10.1093/jxb/erg240 12909687

[B52] Roxrud I. Lid S. E. Fletcher J. C. Schmidt E. D. L. Opsahl-Sorteberg H.-G. (2007). GASA4, One of the 14-member *Arabidopsis* GASA family of small polypeptides, regulates flowering and seed development. Plant Cell Physiol. 48, 471–483. 10.1093/pcp/pcm016 17284469

[B53] Ruokolainen S. Ng Y. P. Broholm S. K. Albert V. A. Elomaa P. Teeri T. H. (2010). Characterization ofSQUAMOSA-like genes inGerbera hybrida, including one involved in reproductive transition. BMC Plant Biol. 10, 128. 10.1186/1471-2229-10-128 20579337 PMC3017819

[B54] Shepard K. A. Purugganan M. D. (2002). The genetics of plant morphological evolution. Curr. Opin. Plant Biol. 5, 49–55. 10.1016/s1369-5266(01)00227-8 11788308

[B55] Shi L. Gast R. T. Gopalraj M. Olszewski N. E. (1992). Characterization of a shoot-specific, GA3-and ABA-regulated gene from tomato. Plant J. 2, 153–159. 10.1046/j.1365-313x.1992.t01-39-00999.x 1302047

[B75] Solano R. Stepanova A. Chao Q. Ecker J. R. (1998). Nuclear events in ethylene signaling: a transcriptional cascade mediated by ETHYLENE-INSENSITIVE3 and ETHYLENE-RESPONSE-FACTOR1. Genes. Dev. 12, 3703–3714. 10.1101/gad.12.23.3703 9851977 PMC317251

[B56] Su M. Huang G. Zhang Q. Wang X. Li C. Tao Y. (2016). The LEA protein, ABR, is regulated by ABI5 and involved in dark-induced leaf senescence in Arabidopsis thaliana. Plant Sci. 247, 93–103. 10.1016/j.plantsci.2016.03.009 27095403

[B57] Szécsi J. Joly C. Bordji K. Varaud E. Cock J. M. Dumas C. (2006). BIGPETALp, a bHLH transcription factor is involved in the control of Arabidopsis petal size. EMBO J. 25, 3912–3920. 10.1038/sj.emboj.7601270 16902407 PMC1553195

[B58] Tähtiharju S. Rijpkema A. S. Vetterli A. Albert V. A. Teeri T. H. Elomaa P. (2011). Evolution and diversification of the CYC/TB1 gene family in Asteraceae—a comparative study in Gerbera (Mutisieae) and sunflower (Heliantheae). Mol. Biol. Evol. 29, 1155–1166. 10.1093/molbev/msr283 22101417

[B59] Uimari A. Kotilainen M. Elomaa P. Yu D. Albert V. A. Teeri T. H. (2004). Integration of reproductive meristem fates by a SEPALLATA-like MADS-box gene. Proc. Natl. Acad. Sci. U.S.A. 101, 15817–15822. 10.1073/pnas.0406844101 15505223 PMC524820

[B60] Varaud E. Brioudes F. Szecsi J. Leroux J. Brown S. Perrot-Rechenmann C. (2011). AUXIN RESPONSE FACTOR8 regulates Arabidopsis petal growth by interacting with the bHLH transcription factor BIGPETALp. Plant Cell. 23, 973–983. 10.1105/tpc.110.081653 21421811 PMC3082276

[B61] Wawrzynska A. Lewandowska M. Sirko A. (2010). Nicotiana tabacum EIL2 directly regulates expression of at least one tobacco gene induced by sulphur starvation. J. Exp. Bot. 61, 889. 10.1093/jxb/erp356 20018902

[B62] Yang Y. Chen B. Dang X. Zhu L. Rao J. Ren H. (2019). Arabidopsis IPGA1 is a microtubule-associated protein essential for cell expansion during petal morphogenesis. J. Exp. Bot. 70, 5231–5243. 10.1093/jxb/erz284 31198941 PMC6793458

[B63] Yin B. Zheng N. Li Y. Tang S. Liang L. Xie Q. (2009). Growth phase-dependent expression of proteins with decreased plant-specific N-glycans and immunogenicity in tobacco BY2 cells. Sci. Chin Ser. C Life Sci. 52, 739–746. 10.1007/s11427-009-0093-5 19727592

[B64] Yoo S.-D. Cho Y.-H. Sheen J. (2007). *Arabidopsis* mesophyll protoplasts: a versatile cell system for transient gene expression analysis. Nat. Protoc. 2, 1565. 10.1038/nprot.2007.199 17585298

[B65] Yu D. Kotilainen M. Pöllänen E. Mehto M. Elomaa P. Helariutta Y. (1999). Organ identity genes and modified patterns of flower development in Gerbera hybrida (Asteraceae). Plant J. 17, 51–62. 10.1046/j.1365-313x.1999.00351.x 10069067

[B67] Zhang L. Li Z. Quan R. Li G. Wang R. Huang R. (2011). An AP2 domain-containing gene, ESE1, targeted by the ethylene signaling component EIN3 is important for the salt response in *Arabidopsis*. Plant Physiol. 157, 854–865. 10.1104/pp.111.179028 21832142 PMC3192559

[B66] Zhang L. Li L. Wu J. Peng J. Zhang L. Wang X. (2012). Cell expansion and microtubule behavior in ray floret petals of Gerbera hybrida: responses to light and gibberellic acid. Photochem Photobiol Sci. 11, 279–288. 10.1039/c1pp05218g 22020373

[B68] Zhang T. Zhao Y. Juntheikki I. Mouhu K. Broholm S. K. Rijpkema A. S. (2017). Dissecting functions of SEPALLATA-like MADS box genes in patterning of the pseudanthial inflorescence of Gerbera hybrida. New Phytol. 216, 939–954. 10.1111/nph.14707 28742220

[B69] Zhao Q. Guo H.-W. (2011). Paradigms and paradox in the ethylene signaling pathway and interaction network. Mol. Plant 4, 626–634. 10.1093/mp/ssr042 21690206

[B70] Zhong S. Zhao M. Shi T. Shi H. An F. Zhao Q. (2009). EIN3/EIL1 cooperate with PIF1 to prevent photo-oxidation and to promote greening of Arabidopsis seedlings. Proc. Natl. Acad. Sci. U.S.A. 106, 21431–21436. 10.1073/pnas.0907670106 19948955 PMC2795496

