# Supplementary material for: An ETHYLENE INSENSITIVE3-LIKE1 Protein Directly Targets the GEG Promoter and Mediates Ethylene-Induced Ray Petal Elongation in Gerbera hybrida
Source: Front Plant Sci. 2020 Jan 24;10:1737. doi: 10.3389/fpls.2019.01737 (PMC6993041; doi:10.3389/fpls.2019.01737)
Supplement: Supplementary file 1 [file DataSheet_1.pdf]

A

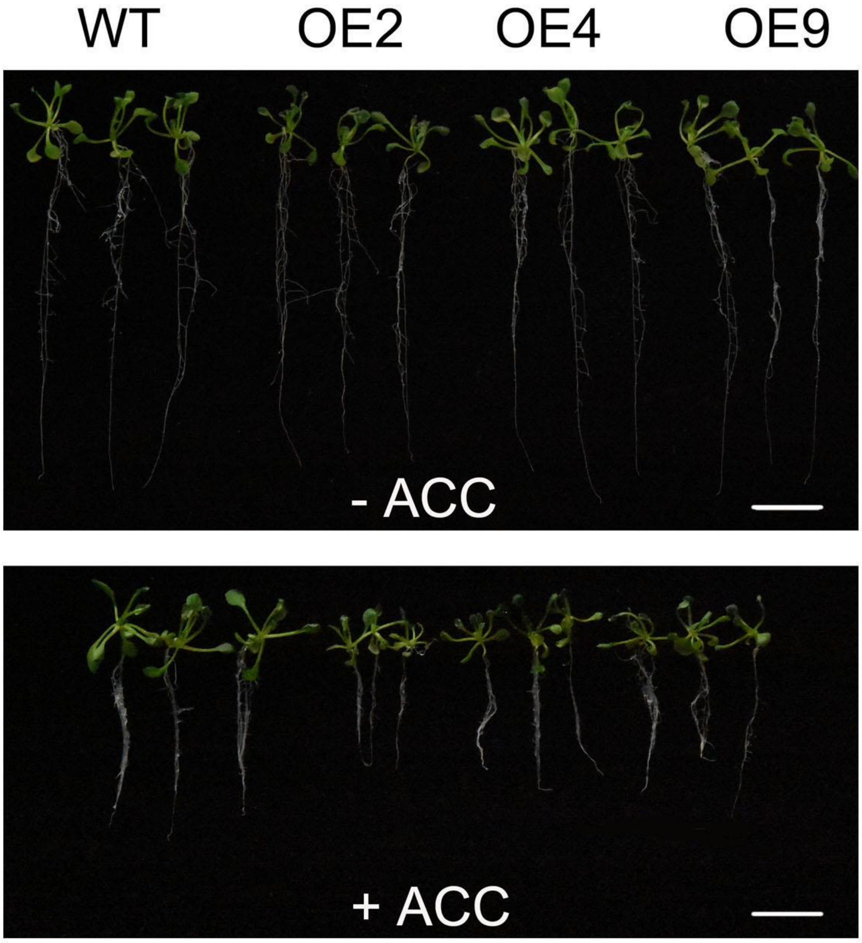
WT
OE2
OE4
OE9
- ACC
+ ACC

B

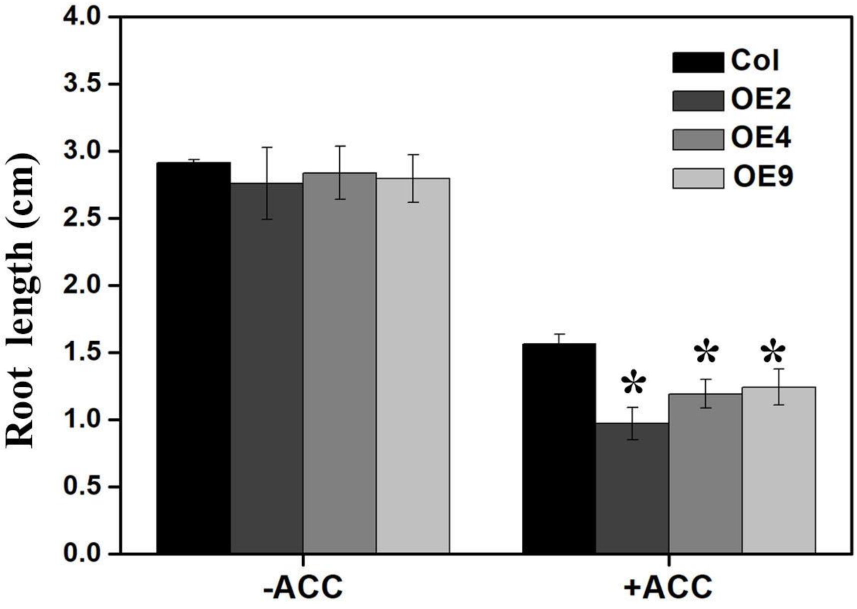
Root length (cm)
4.0
3.5
3.0
2.5
2.0
1.5
1.0
0.5
0.0
Col
OE2
OE4
OE9
*
*
*
-ACC
+ACC

FIGURE S1. Phenotypic characterization of wild-type (WT) and GhEIL1-OE (over-expressing) *Arabidopsis* plants. (A) The phenotype of WT and three different homozygous GhEIL1-OE lines with or without ACC treatments. **(B)** Root length of WT and GhEIL1-OE lines with or without ACC treatments. The seedlings were planted and grown in MS-medium with or without 10 μM ACC for 16 days then observed and measured. Scale bar = 1 cm. Each value is the mean ± SE, n = 30. The significant differences were evaluated by Tukey' HSD test: $*p < 0.05$.

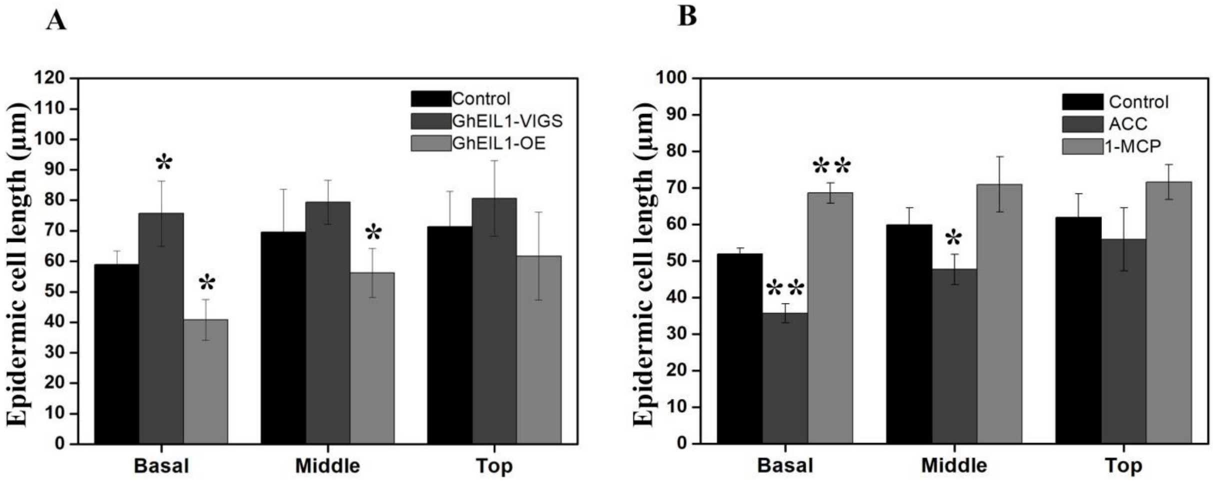

FIGURE S2. Measurement of cell length in the petals after transient transformation and the petals after hormone treatments. (A) The cell length of controls, GhEIL1-VIGS and GhEIL1-OE petals in each region. **(B)** The cell lengthof petals in control, 100 μM ACC and 100 μM 1-MCP treatments in each region. Three biological replicates were analyzed for each measurement. All values indicate means ± SD. Asterisks indicate a significant difference: $*p < 0.05$, $**p < 0.01$, $n > 50$.

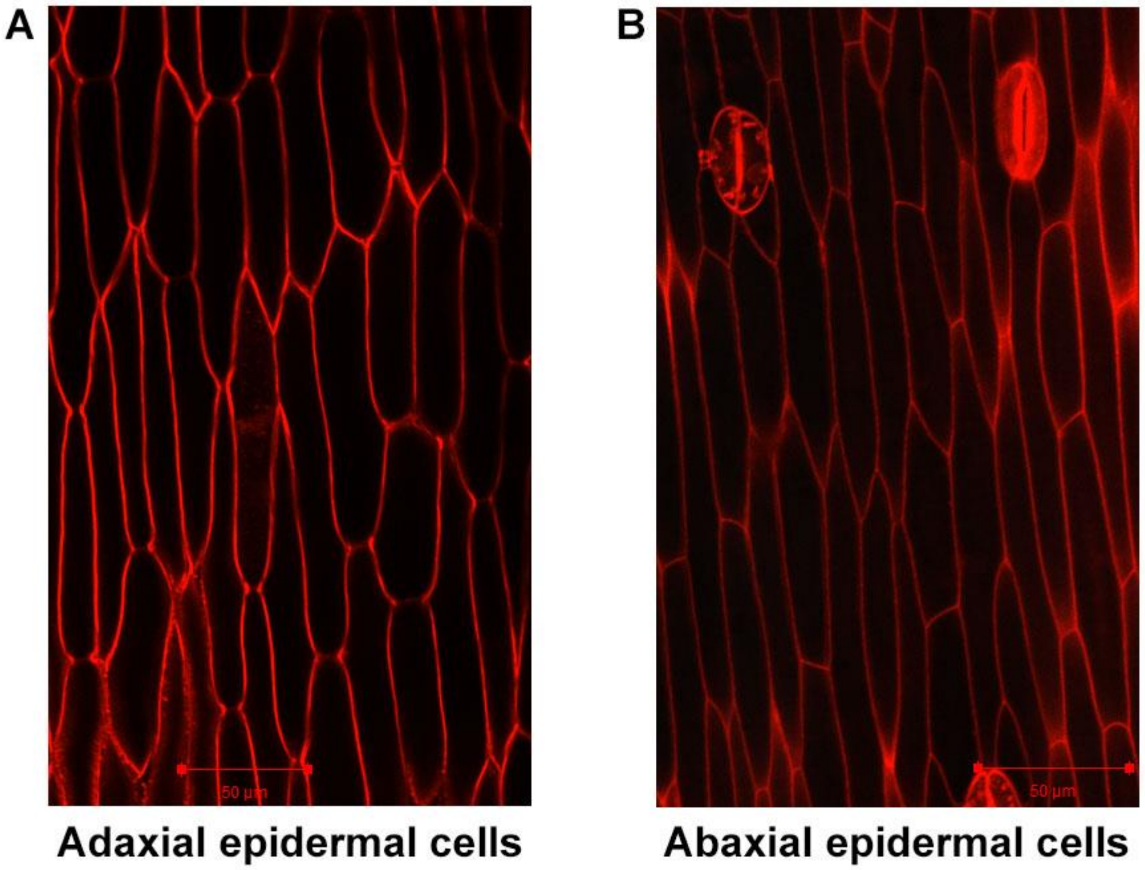

FIGURE S3. Morphological characterization of petal cells. (A) The adaxial epidermal cells in petal. **(B)** The abaxial epidermal cells in petal. Scale bar represents 50 μm.

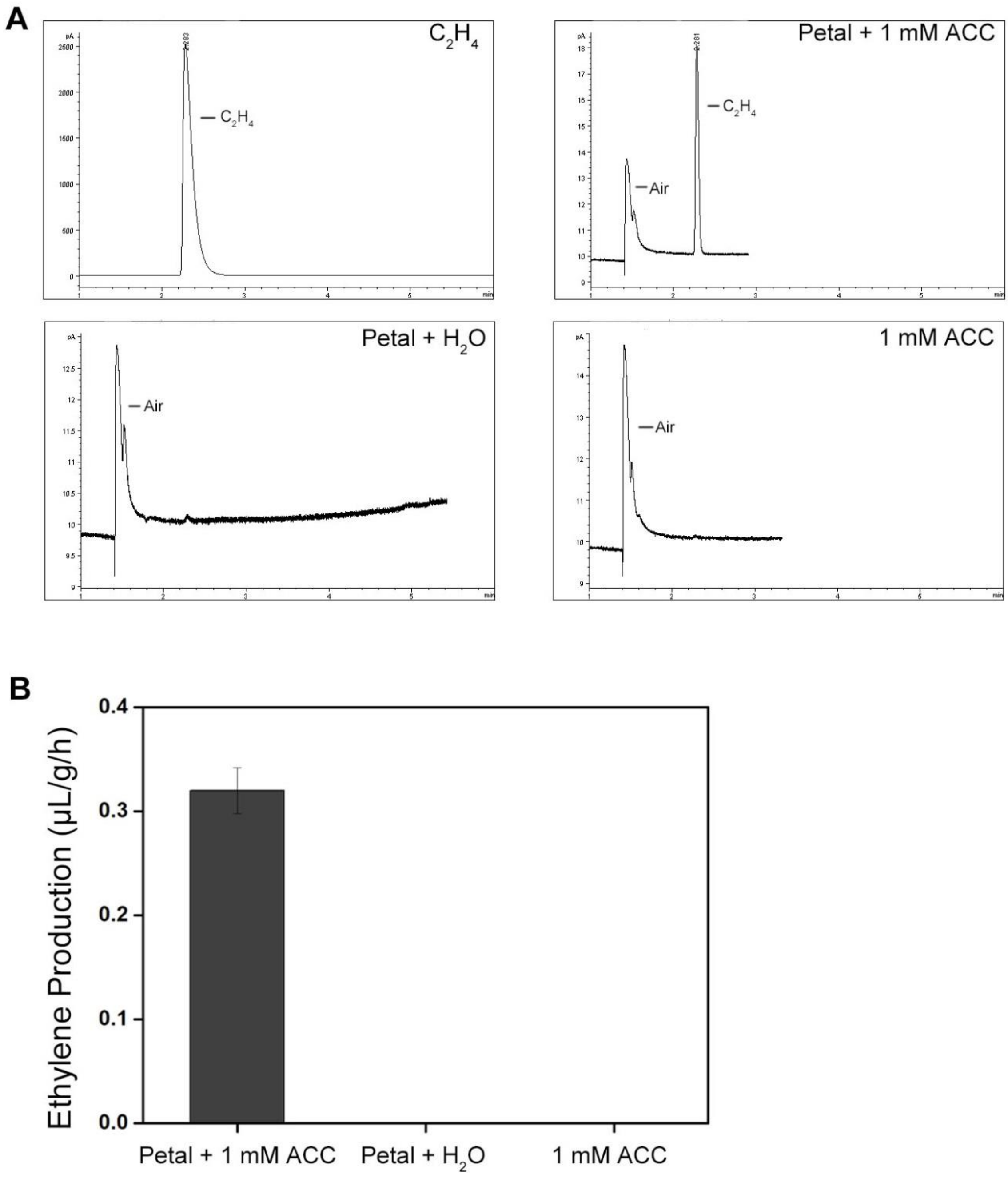

FIGURE S4. Measurement of ethylene content. (A) GC profiles of the gas compounds from different treatments after incubated at 30°C for 1 hour. The petals used here were in stage 3, which were used for hormone treatments. **(B)** The ethylene production in different treatments. The value indicates means ± SD.

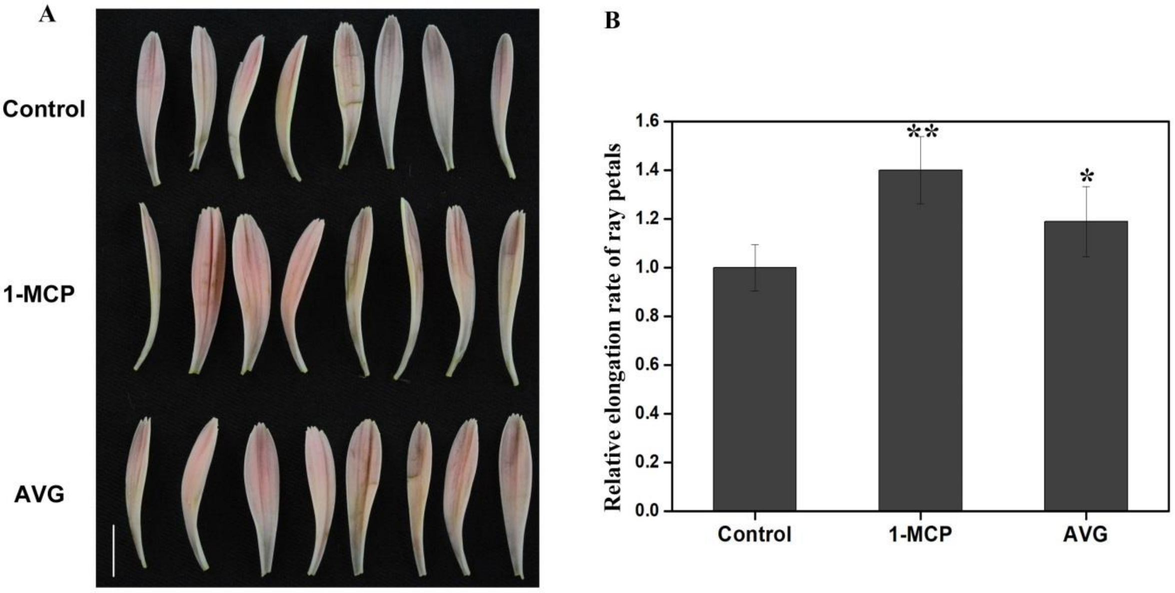

FIGURE S5. The effects of AVG and 1-MCP on petal growth in *G. hybrida*. (A) The phenotypes of petals treated with deionized water (control), AVG and 1-MCP for 7 days. **(B)** The relative elongation rate of ray petals after 7 days treatments. Scale bar represents 1 cm. All values indicate means ± SD. Asterisks indicate a significant difference: $*p < 0.05$, $**p < 0.01$.
