# Supplementary material for: An ETHYLENE INSENSITIVE3-LIKE1 Protein Directly Targets the GEG Promoter and Mediates Ethylene-Induced Ray Petal Elongation in Gerbera hybrida
Source: Front Plant Sci. 2020 Jan 24;10:1737. doi: 10.3389/fpls.2019.01737 (PMC6993041; doi:10.3389/fpls.2019.01737)
Supplement: Supplementary file 2 [file Table_1.pdf]

Supplementary Table S1 List of primer sequences

Primer name	Sequence(5'to 3')
5'-GSP-GhEIL1	GATTACGCCAAGCTTCCAAGTCGCACTCTCTTTTGCCGTC
qRT-PCR-GhEIL1-FP	ATCTGTGCTTGGAGTTCCTGAA
qRT-PCR-GhEIL1-RP	GTACCACATTGAGATATCCTGC
35S-GhEIL1-FP	GGATCCGCATGCTTATGTTTGATGAAATGGGAT
35S-GhEIL1-RP	GAGCTCCTAGTACCACATTGAGATATCCTGCT
pTRV1-FP	TTACAGGTTATTTGGGCTAG
pTRV1-RP	CCGGGTTCAATTCCTTATC
pTRV2-GhEIL1-FP	GAATTCATGGGATTTTGCGAACAACTTA
pTRV2-GhEIL1-RP	GGATCCTAAAGCAGACAATAACGAGCC
pTRV2-FP	TGGGAGATGATACGCTGTT
pTRV2-RP	CCTAAAACTTCAGACACG
EYFP-GhEIL1-FP	CCCGGGATGCTTATGTTTGATGAAATGGGAT
EYFP-GhEIL1-RP	ACTAGTCTAGTACCACATTGAGATATCCTGC
LUC-GhEIL1-GAL4-FP	GGATCCATGCTTATGTTTGATGAAATGGGAT
LUC-GhEIL1-GAL4-RP	TCTAGACTAGTACCACATTGAGATATCCTGC
PBS-GhEIL1-FP	GGATCCATGCTTATGTTTGATGAAATGGGAT
PBS-GhEIL1-RP	TCTAGACTAGTACCACATTGAGATATCCTGC
Y2H-GhEIL1-BD-FP	CGGGATCCCGATGCTTATGTTTGATGAAATGGGAT
Y2H-GhEIL1-BD-RP	GCTGCAGCCTAGTACCACATTGAGATATCCTGC
Y1H-GhEIL1-AD-FP	CCCGGGTATGCTTATGTTTGATGAAATGGGAT
Y1H-GhEIL1-AD-RP	CTCGAGCTAGTACCACATTGAGATATCCTGCT
pGEG975-0800-FP	CTCGAGTTAGACGGCTGATTAAGTGGCCC
pGEG975-0800-RP	GGATCCTTTGTTTTGGGTTGAGTTTGTGC
pGEG402-0800-FP	CTCGAGGTTTACCAACCGTTCCACTGTTACA
pGEG402-0800-RP	GGATCCTTTGTTTTGGGTTGAGTTTGTGC
pGEG468-0800-FP	CTCGAGGTACATTTTATCAACCATTCCGCTG
pGEG468-0800-RP	GGATCCTTTGTTTTGGGTTGAGTTTGTGC
pGEG212-0800-FP	CTCGAGTATTTGCAGTTAAGAGGTCATGAGA
pGEG212-0800-RP	GGATCCTTTGTTTTGGGTTGAGTTTGTGC
pGEG320-0800-FP	CTCGAGGGCAAAGGTTACAATATCATGG
pGEG320-0800-RP	GGATCCCCTTGTTATCCTTTTTATCGAC
pGEG320-mut1-FP	GTTTTAGAGATGAGCCACT
pGEG320-mut1-RP	GTGTTCTTGGGTGACTT
pGEG320-mut2-FP	CACTGTGAGATACTGG
pGEG320-mut2-RP	GAACGGTTGGTAAACTAT
pGEG320-mut3-FP	CATTCCGCTGTCACAAAA
pGEG320-mut3-RP	GTTGATAAAATCTCCTATCC
pGreenII0800-FP	CATTCGCCATTCAGGCTGC
pGreenII0800-RP	TAGCTTCTGCCAACCGAACG
pGEG260-pAbAi-FP	GAGCTCAACCTAAAATTTGTGTTCATATGCA
pGEG260-pAbAi-RP	CTCGAGGGTTGTTATCCTTGTTATCCTTTTT

qRT-PCR-GEG-FP	GCCTTTTCTTGCTTTTGCTCTTC
qRT-PCR-GEG-RP	CGCCTCATCAATCTTGTTCACC
pGEG320-pAbAi-F	AACCTAAAATTTGTGTTCATATGCA
pGEG320-pAbAi-R	GGTTGTTATCCTTGTTATCCTTTTT
